# Impaired natural killer cell migration in HIV-infected individuals is caused by TIGIT-mediated inhibition of HIF-1α-dependent glycolysis

**DOI:** 10.1038/s41419-025-08039-4

**Published:** 2025-11-07

**Authors:** Xiaowen Yu, Jie Zhou, Jie Lei, Hongchi Ge, Zining Zhang, Yajing Fu, Xiaoxu Han, Qinghai Hu, Haibo Ding, Wenqing Geng, Hong Shang, Yongjun Jiang

**Affiliations:** State Key Laboratory for Diagnosis and Treatment of Infectious Diseases, NHC Key Laboratory of AIDS Prevention and Treatment, National Clinical Research Center for Laboratory Medicine, The First Hospital of China Medical University, China Medical University, Shenyang, 110001 China

**Keywords:** HIV infections, NK cells

## Abstract

Natural killer (NK) cells serve as the first line of defense in the immune system and play a crucial role in fighting against HIV infection. The effective function of NK cells is closely related to their migratory capacity. However, the status of NK cell migration in HIV-infected individuals and the underlying regulatory mechanisms remain unknown. Here, we found that NK cell migration was significantly impaired in HIV-infected individuals, with even lower levels in immune non-responders (INRs) compared with immune responders (IRs), and was positively correlated with CD4^+^ T cell counts. Further investigation suggested that the reduced NK cell migration in HIV infection was caused by impaired glycolysis. Mechanistically, NK cell migration was regulated by the HIF-1α pathway. The inhibitory receptor TIGIT suppressed HIF-1α expression by inhibiting PI3K/AKT/mTORC1 and ERK signaling pathways, consequently weakening glycolysis in NK cells of HIV-infected individuals and ultimately leading to downregulation of migration. Collectively, we uncovered a mechanism of reduced NK cell migration during HIV infection and provided new insights for potential immunotherapeutic strategies.

**Figure:**
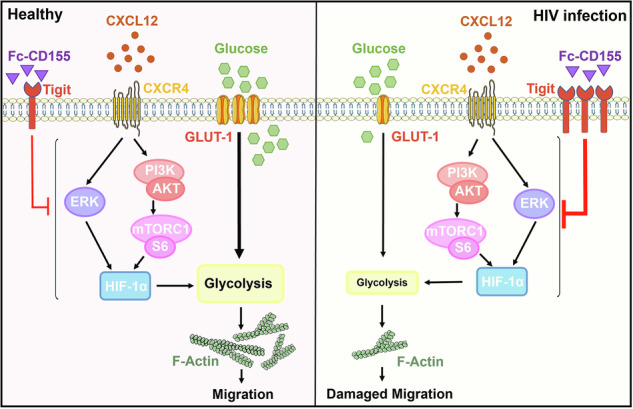
**Schematic of the mechanism of regulating NK cell migration in HC and HIV-infected individuals:** Under normal physiological conditions, NK cells express sufficient levels of the glucose transporter GLUT-1 to support glycolysis, enabling normal glucose uptake. Meanwhile, activation of PI3K/AKT/mTORC1 or ERK signaling pathways induces HIF-1α expression, which subsequently promotes intracellular glycolysis and maintains regular NK cell migration. During HIV infection, the expression of GLUT-1 on NK cells is down-regulated, resulting in impaired glucose uptake. Additionally, High TIGIT expression suppresses HIF-1α expression by inhibiting the PI3K/AKT/ mTORC1 or ERK signaling pathway, thereby impairing glycolysis and ultimately reducing NK cell migration.

**Schematic of the mechanism of regulating NK cell migration in HC and HIV-infected individuals:** Under normal physiological conditions, NK cells express sufficient levels of the glucose transporter GLUT-1 to support glycolysis, enabling normal glucose uptake. Meanwhile, activation of PI3K/AKT/mTORC1 or ERK signaling pathways induces HIF-1α expression, which subsequently promotes intracellular glycolysis and maintains regular NK cell migration. During HIV infection, the expression of GLUT-1 on NK cells is down-regulated, resulting in impaired glucose uptake. Additionally, High TIGIT expression suppresses HIF-1α expression by inhibiting the PI3K/AKT/ mTORC1 or ERK signaling pathway, thereby impairing glycolysis and ultimately reducing NK cell migration.

## Introduction

Acquired immunodeficiency syndrome (AIDS), caused by infection with human immunodeficiency virus (HIV), progressively damages the immune system, leading to opportunistic infections, chronic inflammation, malignant tumors, and ultimately death [1, 2]. Although the mortality among HIV-infected individuals has been greatly reduced with the widespread use of antiretroviral therapy (ART), AIDS is still one of the most serious global public health problems [3].

Natural killer (NK) cells, an important component of the innate immune system, play a crucial role in anti-viral and anti-tumor immunity [4, 5]. As the first line of defense, NK cells are characterized by cytotoxicity and cytokine secretion [6, 7], and their effective function is related to migration capacity [8]. During HIV infection, chemokines such as CCL3, CCL4, and CCL5 secreted by NK cells have been reported to limit viral replication in vitro [9]. Moreover, NK cells can eliminate HIV-infected cells through antibody-dependent cell-mediated cytotoxicity (ADCC), the release of perforin and granzyme, and degranulation marked by CD107a surface expression [10, 11]. Meanwhile, HIV infection leads to a reduction in the number of NK cells, abnormal distribution of cell subsets, disrupted expression of surface markers, and decreased ability of NK cells to lyse virus-infected cells [11–14]. However, NK cell migration in HIV-infected individuals and its relationship with disease progression have yet to be reported.

The chemokine/chemokine receptor axis and cytoskeletal rearrangement exert pivotal roles in the migration of NK cells [15]. The binding of chemokines to their receptors triggers complex signaling cascades that regulate cytoskeletal renewal and induce cell migration [16]. Cytoskeletal rearrangement is conducted by the relative slippage between actin filaments (F-Actin) and myosin [17]. The Actin-binding protein cofilin modifies the actin cytoskeletal architecture by modulating actin polymerization and depolymerization, ultimately promoting cell migration and motility [18–21]. NK cells are known to effectively inhibit tumor cell growth and viral infections, with improved prognosis and increased survival rates in tumor patients related to their higher migration capacity [8, 22]. Impaired NK cell migration to the central nervous system in mice with experimental autoimmune encephalomyelitis (EAE) has been shown to exacerbate disease severity [23]. Therefore, identifying and understanding the changes and regulatory mechanisms of NK cell migration are important in HIV infection.

Accumulating evidence indicates that impaired cellular metabolism is a key factor contributing to NK cell dysfunction in various chronic diseases [24]. It is reported that in mouse NK cells, glucose metabolism is required for NK cell function and cytotoxicity [25, 26]. Additionally, inhibition of glycolysis using 2-deoxy-D-glucose (2-DG) suppresses IFN-γ production and granzyme B expression in NK cells [27, 28]. Cell movement is probably the most energy-consuming cellular activity [29], but the metabolic requirements for NK cell migration have not been explored.

The present study investigated NK cell migration during HIV infection, explored its relationship with HIV disease progression, and found possible causes of impaired NK cell migration in HIV-infected individuals. Then, the signaling pathway and related mechanisms involved in NK cell migration were demonstrated. Finally, this study identified key molecules that may serve as potential targets for recovering NK cell migration in HIV infection. Together, our findings provide novel insights into therapeutic strategies for HIV infection.

## Results

### NK cell migration is impaired in HIV-infected individuals and positively correlated with CD4^+^ T cell counts

**Fig. 1:**
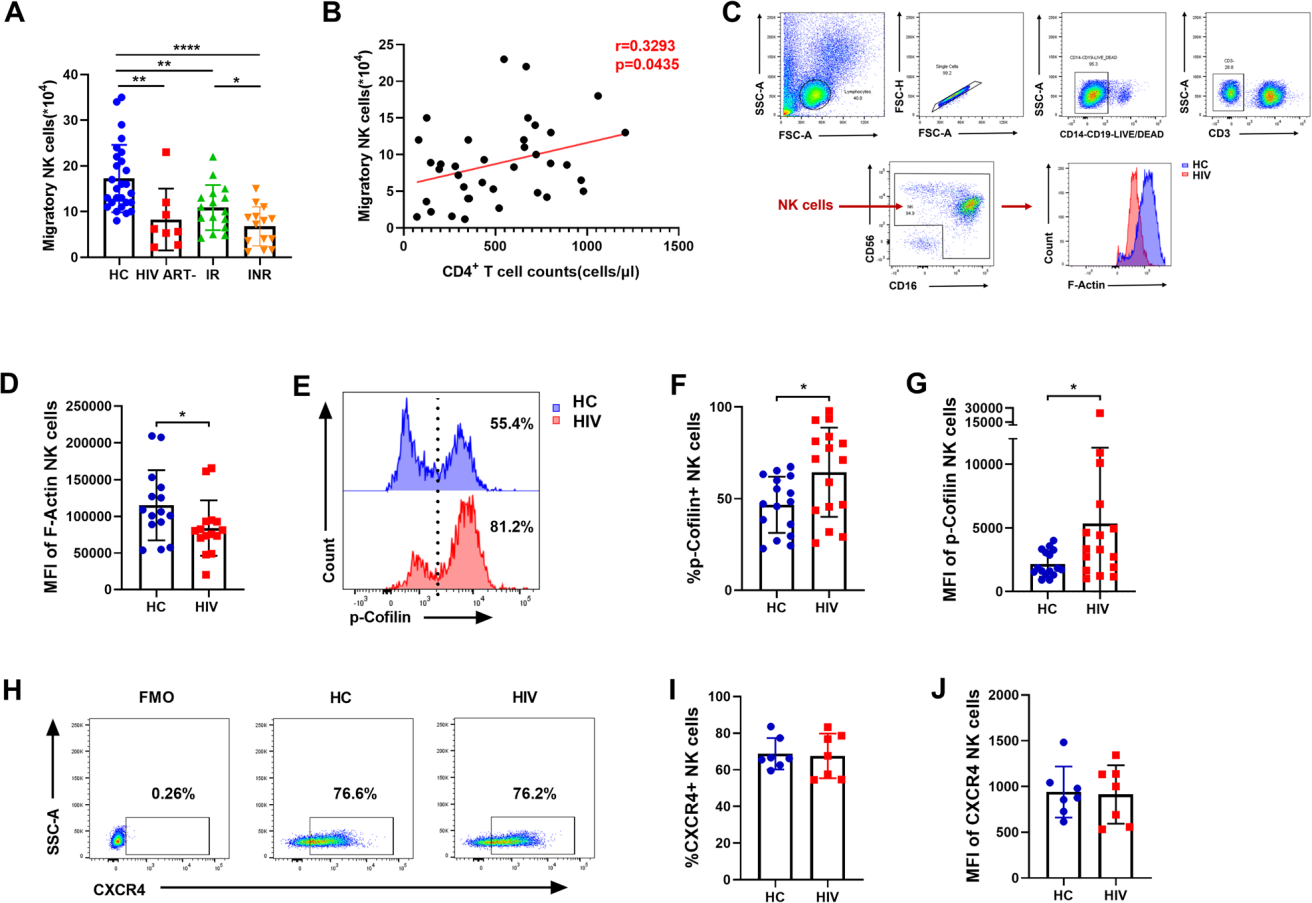
NK cell migration is impaired in HIV infection and positively correlated with CD4^+^ T cell counts. **A** CXCL12 (100 ng/ml)-triggered migration of NK cells in HC individuals (*n* = 27), ART-naïve HIV-infected individuals (*n* = 8), immune responders (IR) (*n* = 16), and immune non-responders (INR) (*n* = 14). *P < 0.05, ***P < 0.001 and ****P < 0.0001 (Kruskal–Wallis test). **B** Correlation between NK cell migration and absolute CD4⁺ T cell counts (cells/μl) in HIV-infected individuals (*n* = 38), using the same samples as analyzed in (**A**), r indicates Spearman rank correlation coefficient. **C**, **D** The flow cytometry gating strategy of NK cells. The MFI of F-Actin in NK cells from the HC (*n* = 15) and HIV (*n* = 15) individuals. **E**–**G** The percentage and MFI of p-cofilin in NK cells from the HC (*n* = 16) and HIV (*n* = 16) individuals. **H**–**J** The percentage and MFI of CXCR4 on NK cells from the HC (*n* = 7) and HIV (*n* = 7) individuals. **D**–**J** *P < 0.05 (Mann–Whitney test).

To investigate alterations in the migratory capacity of peripheral blood NK cells during HIV infection, we assessed NK cell chemotaxis by inducing their transmigration through a microporous membrane in response to chemokine C-X-C motif chemokine ligand 12 (CXCL12). The results showed that CXCL12-mediated NK cell migration was significantly lower in HIV-infected individuals without ART (HIV ART-) (*p* = 0.0036), immune responders (IRs) (*p* = 0.0098), and immune non-responders (INRs) (*p* < 0.0001) compared with HC individuals (Fig. 1A). Moreover, CXCL12-mediated migration of NK cells was lower in INRs than in IRs (*p* = 0.0224). During HIV infection, the absolute CD4^+^ T cell count is a direct indicator of HIV disease progression and immune recovery with ART treatment. Therefore, we further analyzed the correlation between NK cell migration and CD4^+^ T cell counts in HIV-infected individuals. Our analysis revealed that NK cell migration was positively correlated with CD4^+^ T cell counts (Fig. 1B).

We measured the levels of F-Actin and cofilin in NK cells, which are key indicators of cytoskeletal reorganization and cell migration. The results showed that the level of F-Actin in HIV-infected individuals was lower than that in HC individuals (*p* = 0.0367, Fig. 1C, D). The level of Cofilin phosphorylation (inactive) in HIV-infected individuals was higher than that in HC individuals (%: *p* = 0.0387, MFI: *p* = 0.0419, Fig. 1E–G).

To explore whether the impaired migration of peripheral blood NK cells in HIV infection is attributed to the alterations in chemokine receptors, we measured the expression of CXCR4, the receptor for the chemokine CXCL12. Both the percentage and mean fluorescence intensity (MFI) of CXCR4 expression on NK cells were comparable between HIV-infected individuals and HC individuals (Fig. 1H–J), indicating that the impaired migration of NK cells in HIV infection was not caused by altered chemokine receptor expression.

### Impaired glycolysis restricts NK cell migration in HIV-infected individuals

**Fig. 2:**
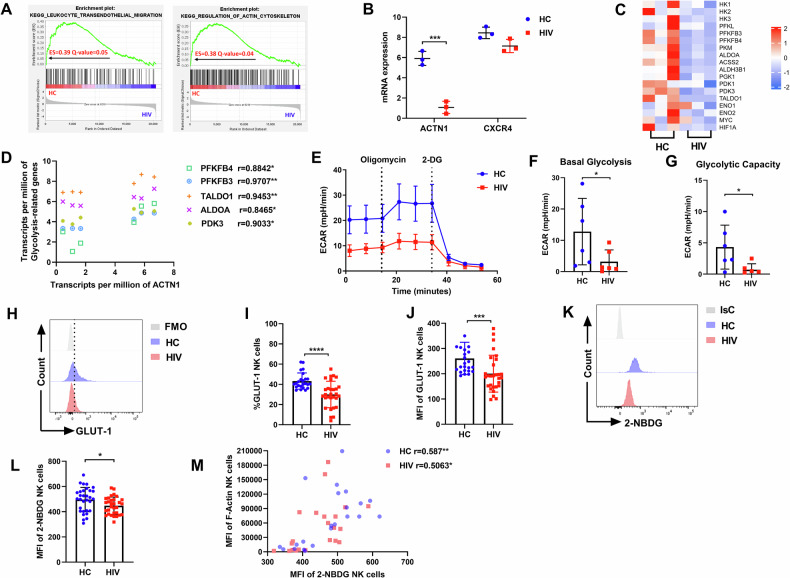
NK cell glycolysis is impaired in HIV infection. **A** GSEA analysis for KEGG lymphocyte trans-endothelial migration and regulation of the Actin cytoskeleton in NK cells from HC and HIV individuals. Positive enrichment score (ES) indicates enrichment in HC individuals. Data from the NCBI Gene Expression Omnibus (GEO) database identifier GSE25669. **B** Comparison of gene expression in HC (*n* = 3) and HIV (*n* = 3) individuals. ***P < 0.001 (Unpaired t test). **C** Heat map shows expression of glycolysis-associated gene expression of NK cells in HIV-infected individuals versus those in HC individuals. **D** The correlation between glycolysis-associated gene expression and *ACTN1* gene expression of NK cells (*n* = 6), r indicates Pearson’s correlation coefficient. **E** ECAR of NK cells in HC and HIV individuals. The basal glycolysis (**F**) and the glycolytic capacity (**G**) of NK cells in HC (*n* = 6) and HIV (*n* = 6) individuals. **H**–**J** The percentage and MFI of GLUT-1 on NK cells from the HC (*n* = 24) and HIV (*n* = 29) individuals. **K**, **L** The MFI of 2-NBDG in NK cells from the HC (*n* = 31) and HIV (*n* = 31) individuals. **F**–**L** *P < 0.05, ***P < 0.001 and ****P < 0.0001 (Mann–Whitney test). **M** The correlation between 2-NBDG and F-Actin of NK cells from the HC (*n* = 22) and HIV (*n* = 22) individuals, r indicates Pearson’s correlation coefficient.

To further explore the factors leading to the impairment of NK cell migration in HIV infection, we analyzed RNA-seq data from HC individuals and HIV-infected individuals obtained from dataset GSE25669 in the GEO database [30]. Gene set-enrichment analysis (GSEA) showed downregulation of genes associated with lymphocyte transendothelial migration and regulation of Actin cytoskeleton in NK cells of HIV-infected individuals compared with HC individuals (Fig. 2A). We also found that *ACTN1* mRNA expression was significantly decreased in HIV-infected individuals, whereas *CXCR4* mRNA expression showed no difference (Fig. 2B), consistent with our observations above.

Subsequently, we found that the levels of glycolysis-related genes in NK cells of HIV-infected individuals were lower than those of HC individuals (Fig. 2C), including hexokinase 1 (*HK1*), hexokinase 2 *(HK2*), 6-phosphofructo-2-kinase/fructose -2,6-bisphosphatase 4 (*PFKFB4*), 6-phosphofructo-2-kinase/fructose-2,6-bisphosphatase 3 (*PFKFB3*), Transaldolase (*TALDO1*), Fructose-bisphosphate aldolase A (*ALDOA*), phosphoglycerate kinase 1 (*PGK1*), pyruvate dehydrogenase kinase 1 (*PDK1*), and pyruvate dehydrogenase kinase 3 (*PDK3*). Moreover, we found that five glycolysis-related genes were significantly positively correlated with *ACTN1* by correlation analysis, including *PFKFB4*, *PFKFB3*, *TALDO1*, *ALDOA*, and *PDK3* (Fig. 2D). *ACTN1* encodes alpha-actin-1, a member of the actin crosslinked protein superfamily, which affects cell adhesion and movement by regulating the actin cytoskeleton [31–33]. Therefore, we speculated that impaired NK cell migration might be associated with altered glycolysis during HIV infection.

To further examine differences in NK cell glycolysis between HC and HIV-infected individuals, we performed a detailed analysis of the extracellular acidification rate (ECAR), which reflects overall glycolytic flux by detecting real-time changes in proton concentration in the media surrounding cells using the Seahorse analyzer. The results showed that the ECAR (Fig. 2E), basal glycolysis (Fig. 2F), and glycolytic capacity (Fig. 2G) of NK cells from HIV-infected individuals were significantly lower than those from HC individuals. Since NK cells rely on glucose uptake to fuel glycolysis [28], we next assessed the expression of the glucose transporter GLUT-1 on the surface of NK cells. The results showed that both the percentage and MFI of GLUT-1 expression were significantly lower in NK cells from HIV-infected individuals compared to those from HC individuals (Fig. 2H–J). Additionally, we evaluated the glucose uptake capacity of NK cells using the fluorescent glucose analog 2-NBDG, which enters cells through glucose transporters and is subsequently phosphorylated by hexokinase, accumulating in the cytoplasm as 2-NBDG-6-phosphate [34, 35]. The results showed that 2-NBDG uptake by NK cells in HIV-infected individuals was lower than that in HC individuals (Fig. 2K, L). To explore whether the cytoskeletal movement of NK cells is related to glycolysis, we examined the correlation between F-Actin and 2-NBDG uptake. We found a significant positive correlation in both HC and HIV-infected individuals (Fig. 2M), indicating that glycolysis may influence the movement of NK cells.

**Fig. 3:**
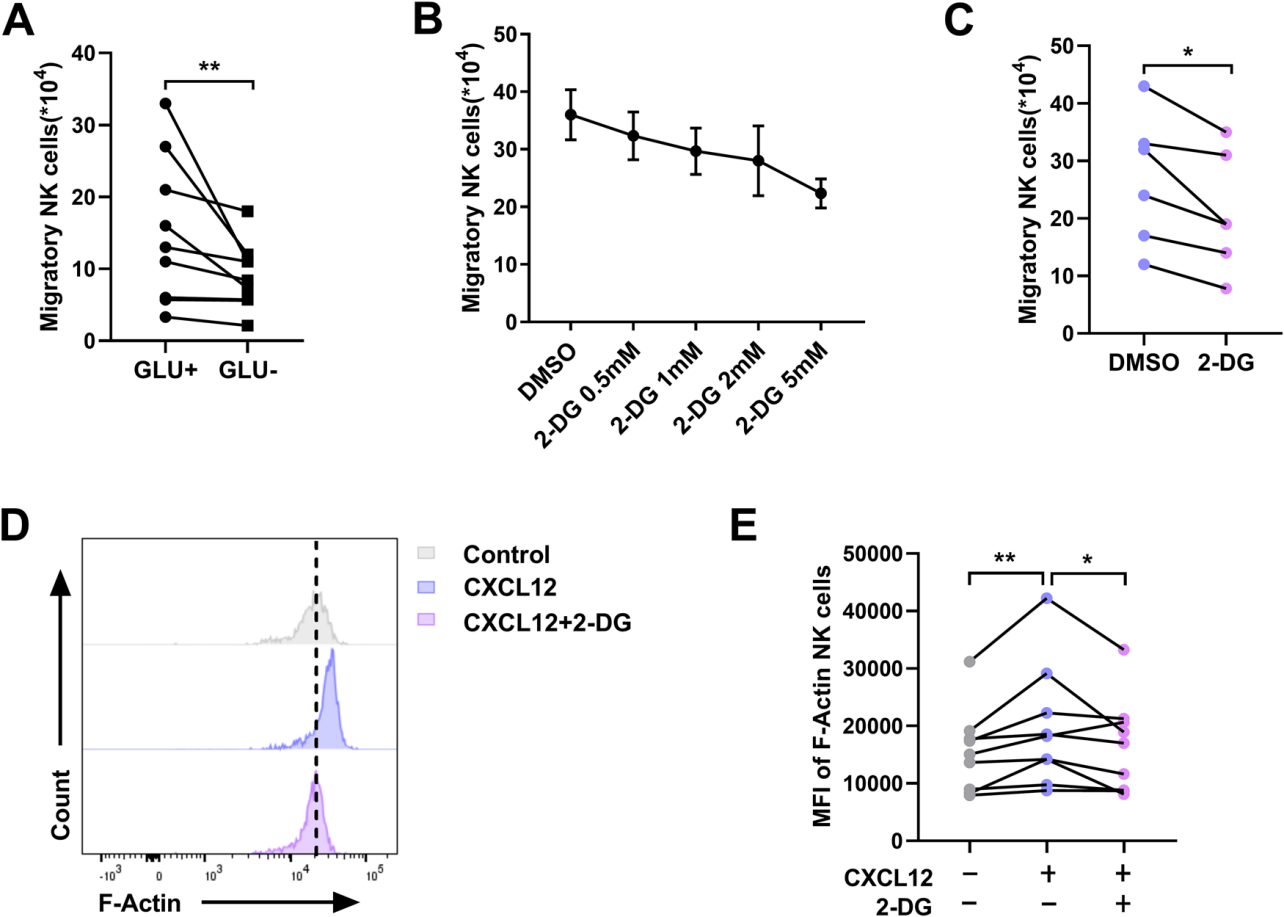
Engagement of glycolysis is necessary for NK cell migration. **A** CXCL12-triggered migration of NK cells in either glucose-containing or glucose-depleted medium (*n* = 9). **B** CXCL12-triggered migration of NK cells treated with different doses of 2-DG (0.5, 1, 2, and 5 mM) or DMSO (*n* = 3). **C** CXCL12-triggered migration of NK cells treated with 2-DG (2 mM) or DMSO (*n* = 6). **A**, **C** *P < 0.05 and **P < 0.01 (Wilcoxon matched-pairs signed rank test). **D** A representative flow cytometry plot demonstrating the effects of 2-DG (2 mM) or DMSO on the levels of F-Actin in CXCL12-stimulated NK cells. **E** The MFI of F-Actin in CXCL12-stimulated NK cells treated with 2-DG (2 mM) or DMSO (*n* = 9). *P < 0.05 and **P < 0.01 (Friedman test).

To investigate whether NK cell migration is regulated by glycolysis, we compared NK cell migration in glucose-containing and glucose-depleted media. As shown in Fig. 3A, glucose depletion limits the migration of NK cells. Moreover, treatment with the glycolysis inhibitor 2-deoxy-D-glucose (2-DG) markedly reduced NK cell migration in a concentration-dependent manner (Fig. 3B, C). In addition, 2-DG treatment suppressed CXCL12-induced F-Actin elevation (Fig. 3D, E). In summary, these results suggest that glycolysis is essential for NK cell migration.

### PI3K/AKT/mTORC1 or ERK signaling pathway is involved in NK cell migration

**Fig. 4:**
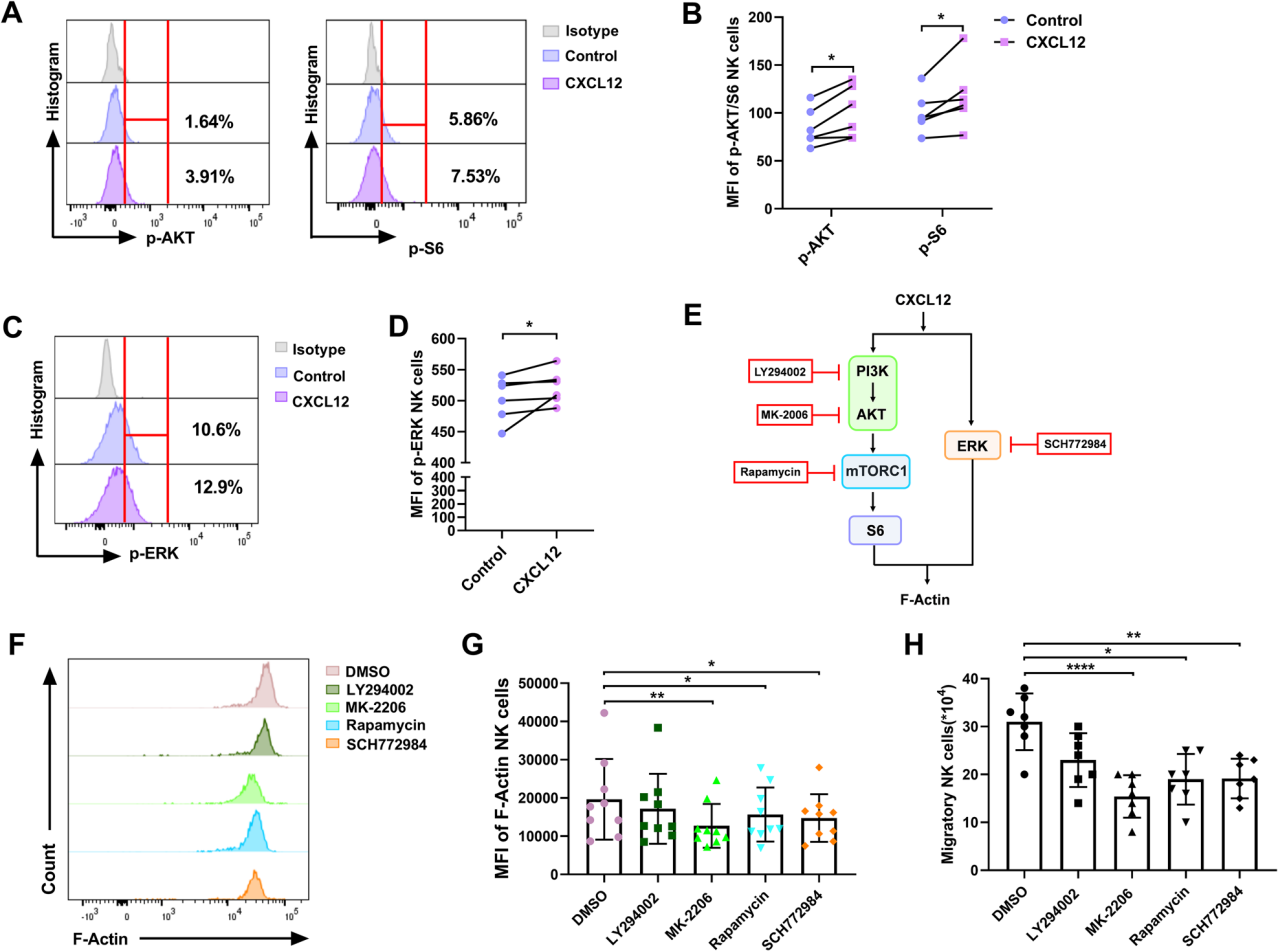
PI3K/AKT/mTORC1 and ERK signaling pathways are involved in NK cell migration. **A** The representative flow cytometry plots show the effects of CXCL12 (100 ng/ml) on the phosphorylation of AKT and S6 in NK cells. **B** The phosphorylation MFI of AKT and S6 in NK cells stimulated with CXCL12 (100 ng/ml) or PBS (Control) (*n* = 6). **C** The representative flow cytometry plots show the effect of CXCL12 (100 ng/ml) on the phosphorylation of ERK in NK cells. **D** The phosphorylation MFI of ERK in NK cells stimulated with CXCL12 (100 ng/ml) or PBS (Control) (*n* = 6). **B**, **D** *P < 0.05 (Wilcoxon matched-pairs signed rank test). **E** Schematic diagram of inhibitors targeting PI3K/AKT/mTORC1 and ERK signaling pathways: LY294002 (PI3K inhibitor), MK-2206 (AKT inhibitor), Rapamycin (mTORC1 inhibitor), and SCH772984 (ERK inhibitor). **F** A representative flow cytometry plot demonstrating the effects of LY294002 (50 μM), MK-2206 (10 μM), Rapamycin (200 nM), SCH772984 (300 nM), or DMSO on the levels of F-Actin in CXCL12-stimulated NK cells. **G** The MFI of F-Actin in CXCL12-stimulated NK cells treated as indicated (*n* = 9). **H** CXCL12-triggered migration of NK cells treated with LY294002 (50 μM), MK-2206 (10 μM), Rapamycin (200 nM), SCH772984 (300 nM) or DMSO (*n* = 7). **G**, **H** *P < 0.05, **P < 0.01, and ****P < 0.0001 (Friedman test).

Based on the above results, we intend to explore the metabolic pathways that link glycolysis to NK cell migration. It has been reported that CXCL12 induces the migration of endothelial cells and bone marrow mesenchymal stem cells via activation of the PI3K/AKT signaling pathway [36, 37]. Additionally, activation of the AKT/mTORC1 signaling pathway has been shown to promote glycolysis and enhance the effector functions of NK cells [28, 38]. Consequently, we hypothesized that the glycolysis-related signaling pathway PI3K/AKT/mTORC1 may be involved in NK cell migration. To verify this conjecture, we assessed the phosphorylation levels of AKT and mTORC1 proteins in CXCL12-treated NK cells. We observed increased levels of phosphorylated AKT and phosphorylated S6 ribosomal protein (pS6), a downstream target and readout of mTORC1 signaling (Fig. 4A, B; Supplementary Fig. 1). In addition, we demonstrated that CXCL12 stimulation also enhanced ERK phosphorylation in NK cells (Fig. 4C, D; Supplementary Fig. 1). To determine if the glycolysis-related PI3K/AKT/mTORC1 or ERK signaling pathways underpin the migration of NK cells, we assessed F-Actin levels and migration capacity in cultured NK cells treated with inhibitors targeting these pathways (Fig. 4E). We found that inhibition of AKT, mTORC1, or ERK significantly reduced both F-Actin levels and NK cell migration (Fig. 4F–H). These results reflect that CXCL12 promotes the migration of NK cells by activating the glycolysis-related PI3K/AKT/mTORC1 and ERK signaling pathways.

### High expression of TIGIT in HIV-infected individuals inhibits glycolysis of NK cells

**Fig. 5:**
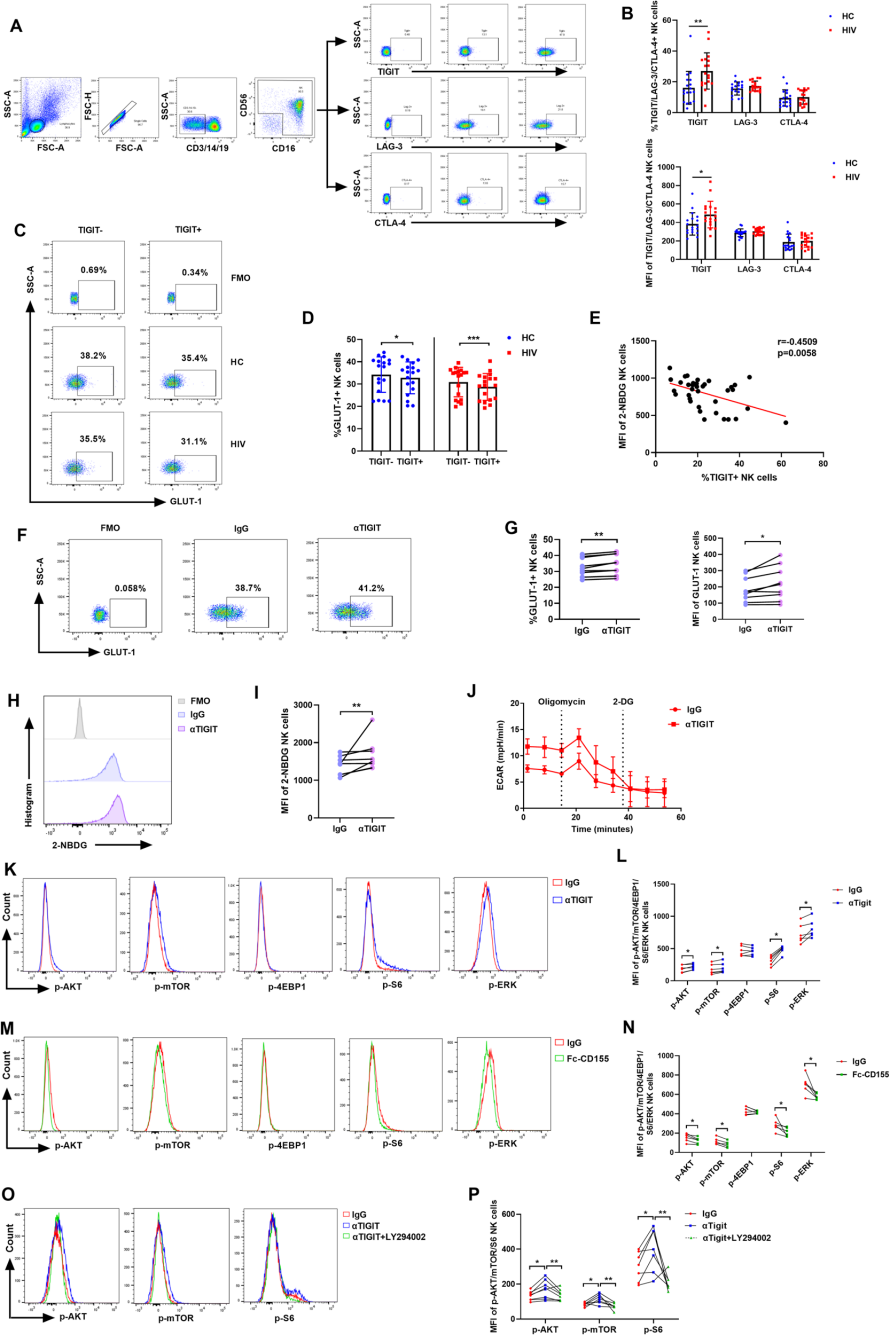
Increased TIGIT-positive NK cells disrupt the glycolysis of HIV-infected individuals by inhibiting PI3K/AKT/mTORC1 or ERK signaling pathways. **A** The representative flow cytometry plots showing the expression of TIGIT, LAG-3 and CTLA-4 on NK cells from HC and HIV individuals. **B** The percentage and MFI of TIGIT, LAG-3, and CTLA-4 on NK cells from HC (*n* = 18) and HIV (*n* = 18) individuals. *P < 0.05 and **P < 0.01 (Mann–Whitney test). **C**, **D** The percentages of GLUT-1 on TIGIT ^-^ or TIGIT ^+^ NK cells from HC (*n* = 18) and HIV (*n* = 18) individuals. *P < 0.05 and ***P < 0.001 (Wilcoxon matched-pairs signed rank test). **E** The correlation between TIGIT and 2-NBDG of NK cells (*n* = 36), r indicates Spearman rank correlation coefficient. **F** The representative flow cytometry plots show the effects of αTIGIT (5 μg/ml) on GLUT-1 expression in NK cells from HIV-infected individuals. **G** The percentage of GLUT-1 on NK cells from HIV-infected individuals treated with αTIGIT (5 μg/ml) or IgG (*n* = 9). **H** The representative flow cytometry plots show the effect of αTIGIT (5 μg/ml) on the glucose uptake in NK cells from HIV-infected individuals. **I** The MFI of 2-NBDG in NK cells from HIV-infected individuals treated with αTIGIT (5 μg/ml) or IgG (*n* = 8). **J** ECAR of NK cells from HIV-infected individuals treated with αTIGIT (5 μg/ml) or IgG. The representative flow cytometry plots show the effects of αTIGIT (5 μg/ml) (**K**) and Fc-CD155 (5 μg/ml) (**M**) on the phosphorylation of AKT, mTOR, 4EBP1, S6, and ERK in NK cells from HIV-infected individuals stimulated with CXCL12 (100 ng/ml). The phosphorylation MFI of AKT, mTOR, 4EBP1, S6, and ERK in NK cells from HIV-infected individuals treated with αTIGIT (5 μg/ml) (**L**) and Fc-CD155 (5 μg/ml) (**N**) (*n* = 6). **G**, **I**, **L**, **N** *P < 0.05 and **P < 0.01 (Wilcoxon matched-pairs signed rank test). **O** The representative flow cytometry plots show the effects of LY294002 (50 μM) on the phosphorylation of AKT, mTOR, and S6 in αTIGIT-treated NK cells from HIV-infected individuals stimulated with CXCL12 (100 ng/ml). **P** The phosphorylation MFI of AKT (*n* = 8), mTOR (*n* = 7), and S6 (*n* = 7) in αTIGIT-treated NK cells from HIV-infected individuals treated with LY294002 (50 μM). *P < 0.05 and **P < 0.01 (Friedman test).

Given that NK cell migration is regulated by glycolysis and glycolysis-related signaling pathways, we then explored the possible reasons for the impaired NK cell glycolysis in HIV-infected individuals. Recent studies have shown that T cell metabolism can be regulated by inhibitory receptors [39, 40], prompting us to investigate whether the reduced glycolysis of NK cells during HIV infection is related to the expression of inhibitory receptors. We assessed the expression of three common inhibitory receptors on NK cells by using flow cytometry. The results showed that the expression of T cell immunoglobulin and ITIM domain (TIGIT) was higher on NK cells from HIV-infected individuals compared to HC individuals, while the expression of lymphocyte-activation gene 3 (LAG-3) and cytotoxic T-lymphocyte-associated protein 4 (CTLA-4) was comparable between the two groups (Fig. 5A, B). Therefore, we further investigated the effect of TIGIT expression on NK cell glycolysis in both HC and HIV-infected individuals. The results showed that the expression of GLUT-1 was significantly lower in TIGIT^+^ NK than TIGIT^-^ NK cells (Fig. 5C, D). Additionally, 2-NBDG uptake was negatively correlated with TIGIT expression in NK cells (Fig. 5E). We next investigated whether TIGIT blockade could restore impaired glycolysis in NK cells during HIV infection. We found that TIGIT blockade enhanced both GLUT-1 expression (Fig. 5F, G; Supplementary Fig. 2A, B) and 2-NBDG uptake (Fig. 5H, I; Supplementary Fig. 2C, D) in NK cells from HIV-infected individuals. Moreover, ECAR levels were increased by blocking TIGIT in NK cells from both HC and HIV-infected individuals (Fig. 5J and Supplementary Fig. 2E).

As the data shown above indicate that TIGIT can inhibit NK cell glycolysis, we continue to explore the underlying mechanisms by which TIGIT regulates this process. Our results above showed that NK cell migration is regulated by the PI3K/AKT/mTORC1 or ERK signaling pathways. Therefore, we examined the effect of TIGIT on the activation of these pathways. As expected, phosphorylation of AKT, mTOR, S6, and ERK was increased in NK cells following TIGIT blockade (Fig. 5K, L; Supplementary Fig. 2F, G). Conversely, treatment with exogenous CD155, a ligand of TIGIT, down-regulated the phosphorylation levels of AKT, mTOR, S6, and ERK (Fig. 5M, N). Moreover, inhibition of PI3K activity decreased the phosphorylation of AKT, mTOR, and S6, even in the presence of TIGIT blockade (Fig. 5O, P). These results suggest that TIGIT inhibits NK cell glycolysis by suppressing the PI3K/AKT/mTORC1 and ERK signaling pathways.

### TIGIT suppresses NK cell migration by inhibiting glycolysis through HIF-1α pathway

**Fig. 6:**
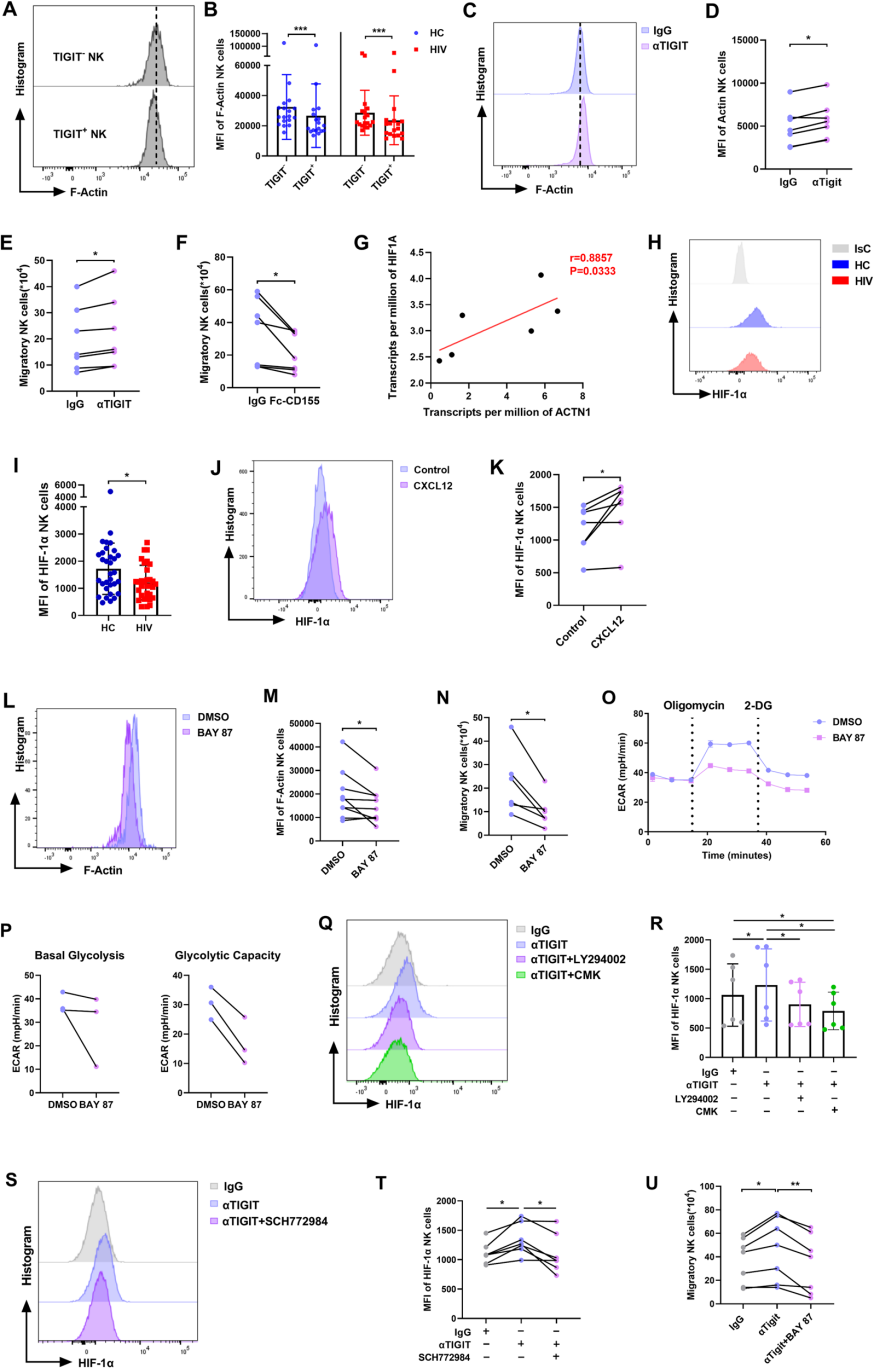
TIGIT suppresses NK cell migration via inhibiting HIF-1α-mediated glycolysis. **A**, **B** The MFI of F-Actin in TIGIT^-^ or TIGIT^+^ NK cells from HC (*n* = 18) and HIV (*n* = 18) individuals. ***P < 0.001 (Wilcoxon matched-pairs signed rank test). **C** A representative flow cytometry plot demonstrating the effects of αTIGIT (5 μg/ml) on the levels of F-Actin in CXCL12-stimulated NK cells from HIV-infected individuals. **D** The MFI of F-Actin in CXCL12-stimulated NK cells from HIV-infected individuals treated with αTIGIT (5 μg/ml) or IgG (*n* = 7). CXCL12-triggered migration of NK cells from HIV-infected individuals treated with αTIGIT (5 μg/ml) (**E**) and Fc-CD155 (5 μg/ml) (**F**) (*n* = 7). **D**–**F** *P < 0.05 (Wilcoxon matched-pairs signed rank test). **G** The correlation between *HIF1A* gene expression and *ACTN1* gene expression of NK cells (*n* = 6). Data from the NCBI Gene Expression Omnibus (GEO) database identifier GSE25669, r indicates Spearman rank correlation coefficient. **H**, **I** The MFI of HIF-1α in NK cells from the HC (*n* = 32) and HIV (*n* = 32) individuals. *P < 0.05 (Mann–Whitney test). **J** A representative flow cytometry plot shows the effects of CXCL12 (100 ng/ml) on the expression of HIF-1α in NK cells. **K** The MFI of HIF-1α in NK cells stimulated with CXCL12 (100 ng/ml) (*n* = 7). **L** A representative flow cytometry plot demonstrating the effects of BAY 87 (10 μM) or DMSO on the levels of F-Actin in CXCL12-stimulated NK cells. **M** The MFI of F-Actin in CXCL12-stimulated NK cells treated with BAY 87 (10 μM) or DMSO (*n* = 9). **N** CXCL12-triggered migration of NK cells treated with BAY 87 (10 μM) or DMSO (*n* = 6). **O** ECAR of NK cells treated with BAY 87 (10 μM) or DMSO. **P** The basal glycolysis and the glycolytic capacity of NK cells treated with BAY 87 (10 μM) or DMSO (*n* = 3). **K**, **M**, **N**, **P** *P < 0.05 (Wilcoxon matched-pairs signed rank test). **Q** A representative flow cytometry plot shows the effects of LT294002 (50 μM) and CMK (50 μM) on the expression of HIF-1α in αTIGIT-treated NK cells from HIV-infected individuals. **R** The MFI of HIF-1α in αTIGIT-treated NK cells from HIV-infected individuals treated with LT294002 (50 μM), CMK (50 μM), or DMSO (*n* = 6). **S** A representative flow cytometry plot shows the effects of SCH772984 (300 nM) on the expression of HIF-1α in αTIGIT-treated NK cells from HIV-infected individuals. **T** The MFI of HIF-1α in αTIGIT-treated NK cells from HIV-infected individuals treated with SCH772984 (300 nM) or DMSO (*n* = 7). **U** CXCL12-triggered migration of NK cells from HIV-infected individuals treated with αTIGIT (5 μg/ml) and BAY 87 (10 μM) (*n* = 7). **R**, **T**–**U** *P < 0.05 and **P < 0.01 (Friedman test).

Based on the above findings that TIGIT weakens the glycolysis of NK cells and that glycolytic inhibition damages the migration of NK cells, we next investigated the role of TIGIT in regulating NK cell migration. We evaluated the expression of F-Actin, a key component involved in the cell migration process. F-Actin Expression was significantly lower in TIGIT ^+^ NK cells compared with TIGIT ^-^ NK cells (Fig. 6A, B). Moreover, TIGIT blockade increased both F-Actin levels and the migration capacity of NK cells from HIV-infected individuals (Fig. 6C–E; Supplementary Fig. 3A, B). Additionally, we observed that the trigger of exogenous CD155 suppressed NK cell migration (Fig. 6F).

To further delve deeper into the mechanism by which TIGIT inhibits NK cell migration, we performed gene-level correlation analysis and found that the mRNA level of *HIF1A* was significantly positively correlated with *ACTN1* (Fig. 6G). We then assessed the protein level of Hypoxia-inducible factor-1 alpha (HIF-1α) in NK cells and found that HIF-1α expression was significantly lower in NK cells from HIV-infected individuals compared to HC individuals (Fig. 6H, I). Furthermore, treatment with CXCL12 significantly increased HIF-1α expression in NK cells (Fig. 6J, K; Supplementary Fig. 3C). We also found that HIF-1α inhibitor BAY 87 significantly reduced F-Actin levels (Fig. 6L, M) and impaired NK cell migration (Fig. 6N). These findings suggest that the HIF-1α pathway contributes to the regulation of NK cell migration.

Moreover, ECAR measurements showed that inhibition of HIF-1α decreased glycolysis of NK cells (Fig. 6O, P). In addition, TIGIT blockade increased HIF-1α expression in NK cells from HIV-infected individuals (Fig. 6Q–T and Supplementary Fig. 3H). In contrast, inhibition of the PI3K/S6K or ERK signaling pathways suppressed HIF-1α expression (Fig. 6Q–T; Supplementary Fig. 3D–G), even in the presence of TIGIT blockade (Fig. 6Q–T and Supplementary Fig. 3H). These results display that TIGIT may suppress NK cell glycolysis through the PI3K/AKT/mTORC1/HIF-1α or ERK/HIF-1α signaling pathways.

Finally, we explored the link between HIF-1α-mediated glycolysis and motility influenced by TIGIT by analyzing the migration of NK cells. Inhibition of HIF-1α activity decreased the migration of NK cells, although TIGIT is blocked (Fig. 6U). These results indicate that TIGIT suppresses NK cell glycolysis and migration, and that HIF-1α inhibition similarly impairs both processes. Moreover, TIGIT downregulates HIF-1α expression in NK cells, suggesting that TIGIT may inhibit glycolysis by suppressing HIF-1α, thereby impairing NK cell migration.

## Discussion

NK cells make an important contribution to resistance to HIV infection, and their function is closely associated with their migration capacity. However, the alterations in NK cell migration and its relationship with disease progression in HIV-infected individuals remain unclear. In the present study, we found that NK cell migration is significantly impaired in HIV-infected individuals compared to HC individuals. Additionally, reduced NK cell migration was correlated with increased disease severity. Vitale et al. reported that the immunosuppressive tumor microenvironment can impair the function and movement of NK cells [41]. In a variety of solid tumors, the NK cell infiltration has been used as a prognostic factor in the survival of tumor patients [42–45]. Salazar-Mather et al. demonstrated that NK cells exert optimal anti-viral defense when recruited to inflammatory sites during murine cytomegalovirus (MCMV) infection [22]. These findings suggest that impaired NK cell migration may hinder the contact between NK cells and infected cells, thus weakening their anti-viral function and potentially reducing the therapeutic outcomes of HIV-infected individuals.

The present study found that NK cell glycolysis is reduced in HIV-infected individuals and that inhibition of glycolysis impairs NK cell migration. Recent studies have highlighted the importance of glycolysis in governing cytokine secretion and cytotoxic activity in NK cells [24, 27, 46–48]. Our findings further demonstrate that NK cell migration is dependent on glycolysis. This is supported by previous reports showing that glycolysis is also essential for the migration of T cells and dendritic cells [49–51]. NK cells primarily utilize glucose as a fuel source for glycolysis, and glucose-driven metabolism enhances their anti-viral and anti-tumor functions [46, 47]. Consistent with this, we found that glucose depletion significantly inhibited NK cell motility. Similarly, Kishore et al. found that glucose depletion inhibited the migration of regulatory T (Treg) cells [50]. Moreover, the cytoskeletal component actin has been shown to interact with glycolytic enzymes [52], forming a glycolytic ATP donor for the ATP-hydrolyzing sodium pump, which provides energy for cytoskeletal rearrangement [53]. Kishore et al. also demonstrated that glucokinase (GCK) promotes cytoskeletal rearrangement and migration of Treg cells by associating with actin [50]. Thus, we hypothesize that glycolysis inhibition restricts cytoskeleton rearrangement and migration of NK cells by suppressing the interaction between glycolytic enzymes and actin. In addition, Haas et al. reported that chemokines upregulate the expression of hexokinase HK1 and pyruvate kinase PKM1/2, the glycolysis rate-limiting enzymes, at both transcriptional and translational levels during CD4^+^ T cell migration. They also demonstrated that chemokine exposure increases glucose transporter gene expression and enhances glycolytic flux in CD4^+^ T cells [49]. Therefore, we speculate that chemokines may similarly induce glycolysis of NK cells by upregulating key glycolytic enzymes or activating glycolysis-related signaling pathways, thereby promoting NK cell migration.

We also observed that the glycolysis-related signaling pathway PI3K/AKT/mTORC1 and ERK are involved in NK cell migration. In various cell types, the PI3K/AKT signaling pathway regulates cellular metabolism, growth, proliferation, actin cytoskeleton rearrangement, and migration [54–56]. The mTOR signaling pathway, a key downstream target of AKT, modulates cellular responses to environmental stress through the formation of two different complexes, mTORC1 and mTORC2 [57]. Kishore et al. reported that pro-migratory stimuli initiate glycolysis in Tregs via the PI3K/AKT/mTORC2 signaling pathway, thereby promoting cytoskeletal rearrangement and cell migration [50]. Moreover, PI3K/AKT/mTOR and ERK signaling pathways have been reported as common downstream effectors of CXCR4 activation [58–61]. Although the metabolic pathways regulating human NK cell migration have not been elucidated previously, Saudemont et al. demonstrated in mice that the PI3K isoforms p110γ and p110δ are essential for mouse NK cell chemotaxis to CXCL12 [62], supporting our findings.

We further investigated the underlying causes of impaired glycolysis in NK cells and found that high TIGIT expression in NK cells from HIV-infected individuals inhibited glycolysis. It is well established that an imbalance between inhibitory and activating receptors contributes to immune cell dysfunction in HIV infection [11, 63]. In recent years, inhibitory receptors have emerged as potential targets for HIV immunotherapy. TIGIT expression varies across different immune cell populations in human peripheral blood, and Wang et al. reported that TIGIT is highly expressed on NK cells, suggesting that it may play a significant role in regulating the effector function of NK cells [64]. Our study demonstrated that TIGIT expression on NK cells from HIV-infected individuals was significantly elevated, consistent with previous research results. In earlier work, we also showed that high TIGIT expression on NK cells inhibited the production of IFN-γ in HIV-infected individuals [65]. Moreover, during HIV infection, TIGIT^+^ NK cells exhibited weaker cytokine secretion capacity compared to TIGIT^-^ NK cells [66], and TIGIT blockade was shown to restore the cytotoxicity of NK cells [67]. However, whether TIGIT affects the metabolism of NK cells has not attracted much attention. Our current study reveals for the first time that TIGIT exerts an inhibitory effect on NK cell glycolysis. Furthermore, we discovered that TIGIT restricts the activation of glycolysis-related pathways in NK cells by reducing the phosphorylation of PI3K/AKT/mTORC1 and ERK signaling pathways. This may be attributed to the binding of TIGIT to CD155, which promotes TIGIT cytoplasmic tail phosphorylation through Src family kinases Fyn and Lck, leading to the recruitment of SHIP-1 (Src homology 2-containing inositol 5-phosphatase 1). Consequently, downstream PI3K/AKT and ERK signaling pathways are inhibited [68–71], resulting in impaired glycolysis in NK cells. Similar to our study, He et al. reported that TIGIT reduces glucose uptake in CD8^+^ T cells by inhibiting the activity of the AKT/mTOR signaling pathway, thereby damaging the cellular metabolism and effector function in gastric cancer [39]. Furthermore, we found that TIGIT inhibits the cytoskeletal rearrangement and migration of NK cells by impairing glycolysis, remarkably, HIF-1α was identified as a key molecule in this process. HIF-1α promotes the gene expression of glucose transporters and glycolytic enzymes, thereby initiating glycolysis [15, 72]. Therefore, we speculated that HIF-1α contributes to NK cell glycolysis, potentially by regulating glucose uptake or the expression of glycolytic enzymes. Moreover, we provided evidence that TIGIT inhibits HIF-1α expression in NK cells through the PI3K/AKT/mTORC1 or ERK signaling pathway. Previous studies have shown that PI3K and AKT signals are involved in promoting HIF-1α synthesis in prostate cancer cells [73], and that mTORC1 enhances glycolytic flux by activating HIF-1α transcription and translation [74, 75]. In addition, inhibition of the ERK/HIF-1α/GLUT-1 pathway has been shown to downregulate macrophage glycolysis in a rat rheumatoid arthritis model [76]. Our study also demonstrated that HIF-1α-mediated glycolysis promotes the migration of NK cells. Liu et al. reported that chemokines activate the HIF-1α signaling pathway in dendritic cells, inducing metabolic reprogramming towards glycolysis and thereby promoting dendritic cell migration [51]. Similarly, Finlay et al. demonstrated that the mTORC1/HIF-1 pathway is necessary for maintaining glycolysis in cytotoxic T lymphocytes (CTL), and that this pathway regulates the expression of essential chemokines and adhesion receptors that mediate T cell trafficking [77]. These findings provide strong support for our conclusions. We also found that blocking TIGIT restored the impaired glycolysis and migration of NK cells in HIV-infected individuals. Together with previous findings that TIGIT blockade can restore the NK cell-mediated immune responses and their cytotoxic activity against the HIV reservoir [66, 67, 78], our results highlight the potential of targeting TIGIT as an immunotherapy strategy for HIV treatment.

In conclusion, our results demonstrate that suppression of glycolysis impairs NK cell migration during HIV infection. This mechanism may be mediated by decreased HIF-1α expression, which is regulated through the PI3K/Akt/mTORC1 and ERK pathways under the control of TIGIT. To the best of our knowledge, this study is the first to investigate the mechanism by which the migration and glycolysis of NK cells are inhibited during HIV infection. These discoveries provide a theoretical basis for the development of immunotherapeutic strategies targeting HIV-infected individuals and may facilitate the identification of therapeutic targets to improve immune function.

## Materials and methods

### Study participants

Peripheral blood was collected from 212 HIV-infected individuals from State Key Laboratory for Diagnosis and Treatment of Infectious Diseases, NHC Key Laboratory of AIDS Prevention and Treatment, The First Affiliated Hospital of China Medical University. HIV-infected individuals included ART-naïve HIV-infected individuals, immunological responders (IRs), and Immunological non-responders (INRs). IRs were HIV-infected individuals on antiretroviral therapy with CD4^+^ T cell counts greater than 500 cells/μl; INRs were those with CD4^+^ T cell counts less than 350 cells/μl. A total of 214 Healthy Control (HC) individuals, matched for age and sex with HIV-infected individuals, were enrolled in this study. Individuals with infection or autoimmune diseases were excluded from the healthy control (HC) group. The study was approved by the Research and Ethics Committee of The First Affiliated Hospital, China Medical University. All subjects provided written informed consent for participation in the study.

### Cell isolation and cell culture

Blood samples were collected from HIV-infected individuals or age- and sex-matched HC individuals. Peripheral blood mononuclear cells (PBMCs) extracted from fresh blood were isolated by Ficoll-Hypaque density gradient centrifugation. Total NK cells were purified from PBMCs by negative selection using the EasySep™ Human NK Cell Enrichment Kit (STEMCELL, Canada). Purified NK cells were cultured in R10 medium (RPMI 1640 medium supplemented with 10% heat-inactivated fetal bovine serum (FBS), penicillin (50 U/ml) (Invitrogen, USA), and streptomycin (50 μg/ml) (Invitrogen, USA)) in the incubator at 37 °C with 5% CO_2_.

NK-92 Cell Line (Pricella, Cat# CL-0530) was screened for mycoplasma contamination and authenticated by STR profiling. NK-92 Cell Line was cultured in α-MEM medium supplemented with Inositol (0.2 mM), β-mercaptoethanol (0.1 mM), Folic Acid (0.02 mM), recombinant IL-2 (200 U/ml), 12.5% HS, 12.5% FBS, and 1% penicillin/streptomycin (Pricella, Cat# CL-0530) in the incubator at 37 °C with 5% CO_2_.

### Transwell migration assay

The migration assay was performed using 24-well Transwell plates containing polycarbonate filters with 8 μm pore size (Corning, NY). The chemokine CXCL12 binds to CXCR4, which is highly expressed by NK cells, to induce NK cell migration [79]. Migration medium (RPMI 1640 supplemented with 10% fetal bovine serum (FBS), 50 U/ml penicillin, and 50 µg/ml streptomycin) or chemokines CXCL12 (100 ng/ml; R&D, USA) were added to the lower chambers. NK cells were added to the upper chamber and incubated at 37 °C for 3 h. Then the upper chamber was removed, and NK cells in the lower chamber were counted by hemocytometer. In some experiments, NK cells were pretreated with DMSO or 2-DG (0.5 mM, 1 mM, 2 mM, 5 mM; Sigma-Aldrich, USA), BAY 87 (10 μM; Cayman, Germany) for 3 h. In other experiments, NK cells were pretreated with Recombinant Human CD155 (5 μg/ml; R&D, USA), anti-TIGIT blocking antibody (5 μg/ml; eBioscience, USA), or IgG control for 1 h.

### Detection of absolute CD4^+^ T cell counts

The TriTEST anti-CD4-FITC/CD8-PE/CD3-PerCP reagents (BD Biosciences, USA), anticoagulated whole blood, and 1× disposable hemolysin were added to the Trucount tubes for a single-platform lyse-no-wash procedure. The cell counts were detected by flow cytometer (BD FACS Calibur), and the results were analyzed by MultiSET software.

### Measurement of F-Actin and phosphorylated cofilin

NK cells were resuspended in BD Cytofix/Cytoperm Solution (BD Biosciences, USA) for 30 min at 4 °C in the dark, then washed 2 times with 1× BD Perm/Wash™ buffer (BD Biosciences, USA). F-Actin was labeled with Alexa Fluor 647 Phalloidin (Thermo Fisher Scientific, Cat# A22287, 1:200 dilution) for 30 min at 4 °C in the dark. Then the cells were washed with BD Perm/Wash Buffer and detected by flow cytometry. In some experiments, NK cells were incubated for 16 h at 37 °C with the following inhibitors purchased from Sigma-Aldrich, MCE or Cayman: 2-DG (5 mM), Rapamycin (200 nM), LY294002 (50 μM), MK-2206 (10 μM), SCH772984 (300 nM), BAY 87 (10 μM). Then the cells were stimulated with CXCL12 (100 ng/ml) at 37 °C for 15 min. After treatment of NK cells with CXCL12, cells were washed with cold phosphate-buffered saline (PBS) with 2% fetal bovine serum and then stained by flow cytometry. BD Horizon™ Fixable Viability Stain 620 (BD Biosciences, USA) was used to evaluate cell viability.

Cofilin dynamics were measured as the percentage and mean fluorescence intensity (MFI) of phospho-cofilin. NK cells were fixed with 1.5% formaldehyde and incubated at room temperature for 10 min. Then the cells were permeabilized with cold (−20 °C) methanol on ice for 10 min. The cells were next stained with rabbit anti-p-cofilin (Ser3) antibody (clone 77G2, 1:100 dilution, Cell Signaling Technology) for 1 h and washed 2 times. The cells were labeled with Alexa Fluor 488-conjugated chicken anti-rabbit antibody (Cat# A2144, 1:200 dilution, Invitrogen) for 30 min and then detected by flow cytometry.

### Cell surface and intracellular staining

The phenotype of NK cells was analyzed by flow cytometry using freshly isolated PBMCs. CXCR4 and GLUT-1 expression on NK cells were detected using the following fluorophore-conjugated antibodies: FITC anti-human CD3 (1:50, clone UCHT1, BD Biosciences), Percp-Cy5.5 anti-human CD14 (1:50, clone M5E2, BD Biosciences), Percp-Cy5.5 anti-human CD19 (1:50, clone HIB19, BD Biosciences), PE-Cy7 anti-human CD56 (1:30, clone B159, BD Biosciences), APC-Cy7 anti-human CD16 (1:30, clone 3G8, BD Biosciences), BV421 anti-human CXCR4 (1:20, clone 12G5, BioLegend), and PE anti-human GLUT-1(1:10, clone 202915, R&D). TIGIT, LAG-3, and CTLA-4 expression on NK cells were detected using the following fluorophore-conjugated antibodies: Percp-Cy5.5 anti-human CD3/CD14/CD19, PE-Cy7 anti-human CD56, APC-Cy7 anti-human CD16, APC anti-human CTLA-4 (1:30, clone L3D10, Biolegend), APC Mouse IgG2a κ isotype control (1:30, clone MOPC-21, Biolegend), BV421 anti-human TIGIT (1:30, clone A15153G, Biolegend), BV421 Mouse IgG2a κ isotype control (1:30, clone MOPC-173, Biolegend), FITC anti-human LAG-3 (1:30, clone 7H2C65, Biolegend), and FITC Mouse IgG1 κ isotype control (1:30, clone MOPC-21, Biolegend). In brief, freshly isolated PBMCs were labeled with the corresponding fluorescently antibodies cocktails for 30 min at 4 °C. Then, stained cells were washed with PBS containing 2% FBS and detected by LSR II flow cytometer (BD Biosciences). The data was analyzed by FlowJo v10 software (Ashland, OR, USA).

For intracellular HIF-1α staining, eBioscience™ Foxp3/Transcription Factor Staining Buffer Set was used (Thermo Fisher Scientific, USA). Cells were fixed/permeabilized for 45 min at 4 °C with the prepared Fixation/Permeabilization working solution, which was prepared by mixing 1 part of fixation/permeabilization concentrate with 3 parts of fixation/permeabilization diluent. Cells were then washed twice with 1× permeabilization buffer and stained with PE anti-human HIF-1α antibody (1:10, clone 241812, R&D) for 45 min at 4 °C. The cells were finally washed with 1× permeabilization buffer, then centrifuged and resuspended in 200 μl PBS.

### Glucose uptake assay

2-NBDG (2-(N-(7-nitrobenzene-2-oxy-1, 3-diazole-4-yl) amino) -2-deoxyglucose) is a fluorescent glucose analogue used to monitor glucose uptake in cells. According to the manufacturer’s instructions, freshly isolated cells were resuspended in glucose-free R10 with 2-NBDG (Invitrogen, Cat# N13195) at a final concentration of 100 μM for 30 min at 37 °C. Finally, the cells were washed twice with warm PBS, and stained for surface markers at 4 °C for 30 min in a dark environment. The absorption of fluorescence by NK cells was detected immediately by LSR II flow cytometry.

### Extracellular acidification rate assays

A Seahorse XF HS Mini Analyzer (Agilent, USA) was used for real-time analysis of the extracellular acidification rate (ECAR) of purified NK cells cultured for 48 h in 10 ng/ml IL-12, 50 ng/ml IL-15, and 100 ng/ml IL-18 (all from R&D, USA). In some experiments, NK cells were pretreated with anti-TIGIT blocking antibody (5 μg/ml; eBioscience, USA) for 1 h prior to stimulation. Briefly, 4 × 10^5^ treated NK cells were resuspended in Seahorse XF DMEM Medium containing glutamine (1 mM; Agilent, USA) and seeded into an 8-well XF cell culture microplate (Agilent, USA). All cell culture plates were treated with Cell-Tak (Corning, USA) for 20 min to ensure that NK cells adhered to the culture plates. Measurements of ECAR following the addition of oligomycin (2 μg/ml; MCE, USA) and 2-deoxyglucose (50 mM; Agilent, USA) allowed for the calculation of basal glycolysis and glycolytic capacity. Each experimental sample was done in at least triplicate wells.

### Phosphorylation detection of signaling pathways

Freshly isolated NK cells were stimulated with or without chemokine CXCL12 (100 ng/ml; R&D, USA) for 15 min at 37 °C and 5% CO_2_. Alternatively, NK cells were stimulated with 100 ng/ml CXCL12 (R&D, USA) for 15 min after treatment with 5 μg/ml anti-TIGIT blocking antibody (5 μg/ml; eBioscience, USA) for 1 h. Then the cells were immediately fixed to stop stimulation with the same volume of warm BD Phosflow™ Fix Buffer I (BD Biosciences, USA) for 10 min at 37 °C. Cells were washed with PBS (2%FBS) and resuspended in 100 ul ice-cold BD Phosflow™ Perm Buffer III (BD Biosciences, USA) and incubated on ice for 30 min. Subsequently, the cells were washed twice with PBS (2%FBS) and stained for Alexa 647-AKT (pS473)(1:20, clone M89-61, BD Biosciences), APC-S6 (Ser235, Ser236)(1:20, clone cupk43k, eBioscience), PE-mTOR (pS2448)(1:20, clone O21-404, BD Biosciences), Alexa 488-4EBP-1 (pT69)(1:5, clone M34-273, BD Biosciences), and Alexa 488-ERK1/2 (pT202/pY204)(1:5, clone 20A, BD Biosciences) for 30 min at 4 °C. Finally, the cells were washed and resuspended in PBS (2%FBS) and immediately analyzed by LSR II flow cytometry.

### Western blotting

Whole-cell lysates were lysed in RIPA lysis buffer (Beyotime, Cat# P0013B), Phosphatase inhibitor cocktail (Beyotime, Cat# P1081), and protease inhibitor cocktail (Thermo Fisher Scientific, Cat# 87785). Equivalent amounts of protein, as determined by BCA protein assay (Beyotime, Cat# 5000001), were separated by SDS (Thermo Fisher Scientific, Cat# NP0002) and transferred to nitrocellulose membrane (Thermo Fisher Scientific, Cat# PB3310). Membranes were blocked for 1 h at room temperature in SuperBlock blotting blocking buffer (Thermo Fisher Scientific, Cat# 37517) and were incubated at 4 °C for 16 h in a dark environment with the primary antibodies (HIF-1α 1:1000, clone D1S7W; p-AKT (Ser473) 1:1000 dilution, Rabbit Polyclonal; AKT 1:1000 dilution, Rabbit Polyclonal; p-S6 (Ser240/244) 1:1000 dilution, Rabbit Polyclonal; S6 1:1000 dilution, clone 5G10; Phospho-p44/42 MAPK (Erk1/2) (Thr202/Tyr204) 1:2000 dilution, clone D13.14.4E; p44/42 MAPK (Erk1/2) 1:1000 dilution, clone L34F12; all from Cell Signaling Technology) and beta Actin (1:5000 dilution; Thermo Fisher Scientific). HRP-conjugated secondary antibody (1:5000 dilution; Thermo Fisher Scientific) was subsequently added. Films were then developed. The band intensity was analyzed using ImageJ (NIH).

### TIGIT blockade and activation assays

TIGIT was blocked by anti-TIGIT blocking antibody (αTIGIT, 5 μg/ml; eBioscience, USA) and activated by recombinant human CD155 (Fc-CD155, 5 μg/ml; R&D, USA). The effects of blocking and activating TIGIT on NK cells were assessed by pre-incubating cells in the presence of αTIGIT (5 μg/ml; eBioscience, USA) or Fc-CD155 (5 μg/ml; R&D, USA) for 1 h, followed by stimulation with CXCL12 (100 ng/ml; R&D, USA) for 15 min or 24 h at 37 °C and 5% CO_2_. After incubation, signaling pathway phosphorylation, GLUT-1 expression, and 2-NBDG uptake were evaluated as described above. For F-Actin detection, NK cells were cultured with αTIGIT antibody (5 μg/ml; eBioscience, USA) for 1 h, and then stimulated with CXCL12 (100 ng/ml; R&D, USA) at 37 °C and 5% CO_2_ for 15 min.

### Online database analysis

We use the online resource GEO database (https://www.ncbi.nlm.nih.gov/geo/) to analyze the difference of NK cell RNA-sequencing (RNA-seq) data between HC individuals and HIV-infected individuals. The RNA-seq data set was from GEO with the number of GSE25669. TPM (transcripts per million) was used to measure gene expression levels. The data were normalized to TPM using the “limma” package in R v3.6.3 software (https://www.r-project.org/), and Log-2 transformations were performed on all data. GSEA was analyzed by GSEA v4.1.0 software (Broad Institute software). The enrichment pathways data set was analyzed using the Kyoto Encyclopedia of Genes and Genomes (KEGG) in the MSigDB data set. Heat map was plotted using phatmap and ComplexHeatmap package in R.

### Statistical analysis

All flow cytometry data were analyzed by FlowJo software v10.6.2. Statistical analysis was performed using GraphPad Prism v8.0 software and SPSS Statistics v26.0 software programs. The results are presented as mean values ± standard deviation (SD) unless otherwise indicated. Two groups of data were analyzed by Wilcoxon matched-pairs signed rank tests or Mann–Whitney test. Kruskal–Wallis test or Friedman test was used to compare the data of multiple groups. Spearman rank correlation test was used for correlation analysis. *P*-value of less than 0.05 was considered to be significant (*P < 0.05, **P < 0.01, ***P < 0.001, ****P < 0.0001). All statistical tests were two-sided.

## Supplementary information

supplementary Figure legends

Supplementary Figure 1

Supplementary Figure 2

Supplementary Figure 3

Full and uncropped western blots

## Data Availability

The RNA-seq dataset analyzed in this study was obtained from the GEO repository under accession number GSE25669. All relevant data are available from the corresponding author on reasonable request.
