# Supplementary material for: Impaired natural killer cell migration in HIV-infected individuals is caused by TIGIT-mediated inhibition of HIF-1α-dependent glycolysis
Source: Cell Death Dis. 2025 Nov 7;16(1):805. doi: 10.1038/s41419-025-08039-4 (PMC12594964; doi:10.1038/s41419-025-08039-4)
Supplement: Supplementary file 1 — supplementary Figure legends [file 41419_2025_8039_MOESM1_ESM.docx]

**Supplementary Figure 1.** NK-92 cells were stimulated with CXCL12 (100 ng/ml) or a cytokine cocktail containing IL-12 (10 ng/ml), IL-15 (50 ng/ml), and IL-18 (100 ng/ml). Phosphorylation of AKT, S6, and ERK was quantified by Western blotting.

**Supplementary Figure 2.** **(A and B)** The percentage and MFI of GLUT-1 on NK cells from HIV-infected individuals treated with αTIGIT (5 μg/ml), IgG, or untreated (Control) (n = 8). **(C and D)** The MFI of 2-NBDG in NK cells from HIV-infected individuals treated with αTIGIT (5 μg/ml), IgG, or untreated (Control) (n = 8). **(B and D)** **P* < 0.05 and ***P* < 0.01 (Wilcoxon matched-pairs signed rank test). **(E)** ECAR of NK cells from HC individuals with or without αTIGIT (5 μg/ml) treatment (Control). **(F and G)** NK-92 cells were treated with αTIGIT (5 μg/ml) or left untreated. Phosphorylation of AKT, S6 (F), and ERK (G) was quantified by Western blotting.

**Supplementary Figure 3.** **(A and B)** The MFI of F-Actin in CXCL12-stimulated NK cells from HIV-infected individuals treated with αTIGIT (5 μg/ml), IgG, or untreated (Control) (n = 8). **(C)** NK-92 cells were treated with CXCL12 (100 ng/ml) or left untreated. HIF-1α Protein expression was quantified by Western blotting. **(D and E)** The MFI of HIF-1α in NK cells treated with LT294002 (50 μM), CMK (50 μM) or DMSO (n = 6). **P* < 0.05 and ***P* < 0.01 (Friedman test). **(F and G)** The MFI of HIF-1α in NK cells treated with SCH772984 (300 nM) or DMSO (n = 6). ***P* < 0.01 (Wilcoxon matched-pairs signed rank test). **(H)** NK-92 cells treated with αTIGIT were incubated with LY294002 (50 μM), CMK (50 μM), SCH772984 (300 nM), or DMSO. HIF-1α protein expression was detected by Western blotting.
