# Supplementary figures and images for: Impaired natural killer cell migration in HIV-infected individuals is caused by TIGIT-mediated inhibition of HIF-1α-dependent glycolysis
Source: Cell Death Dis. 2025 Nov 7;16(1):805. doi: 10.1038/s41419-025-08039-4 (PMC12594964; doi:10.1038/s41419-025-08039-4)

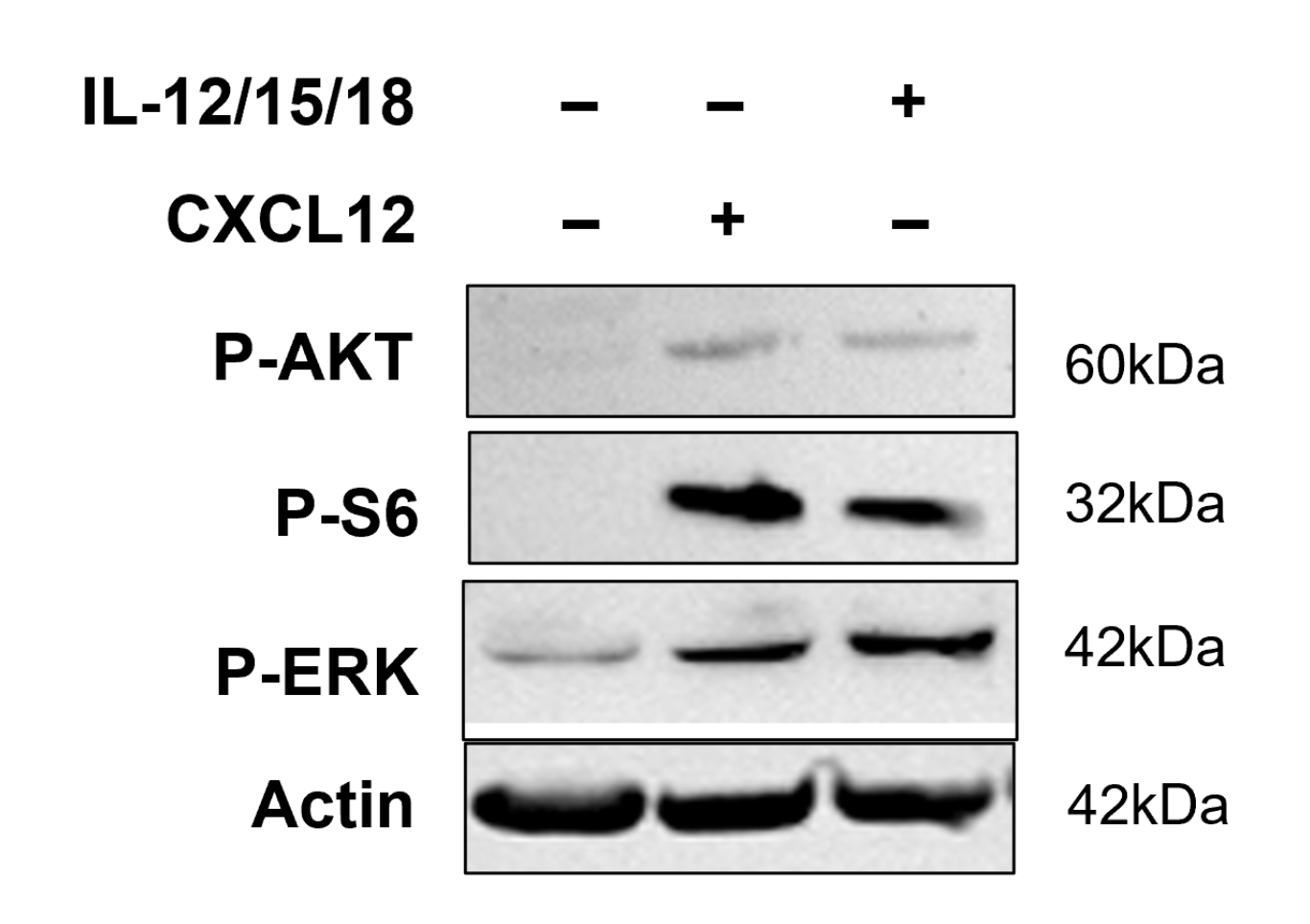

Supplement: Supplementary file 2 — Supplementary Figure 1 [file 41419_2025_8039_MOESM2_ESM.tif]

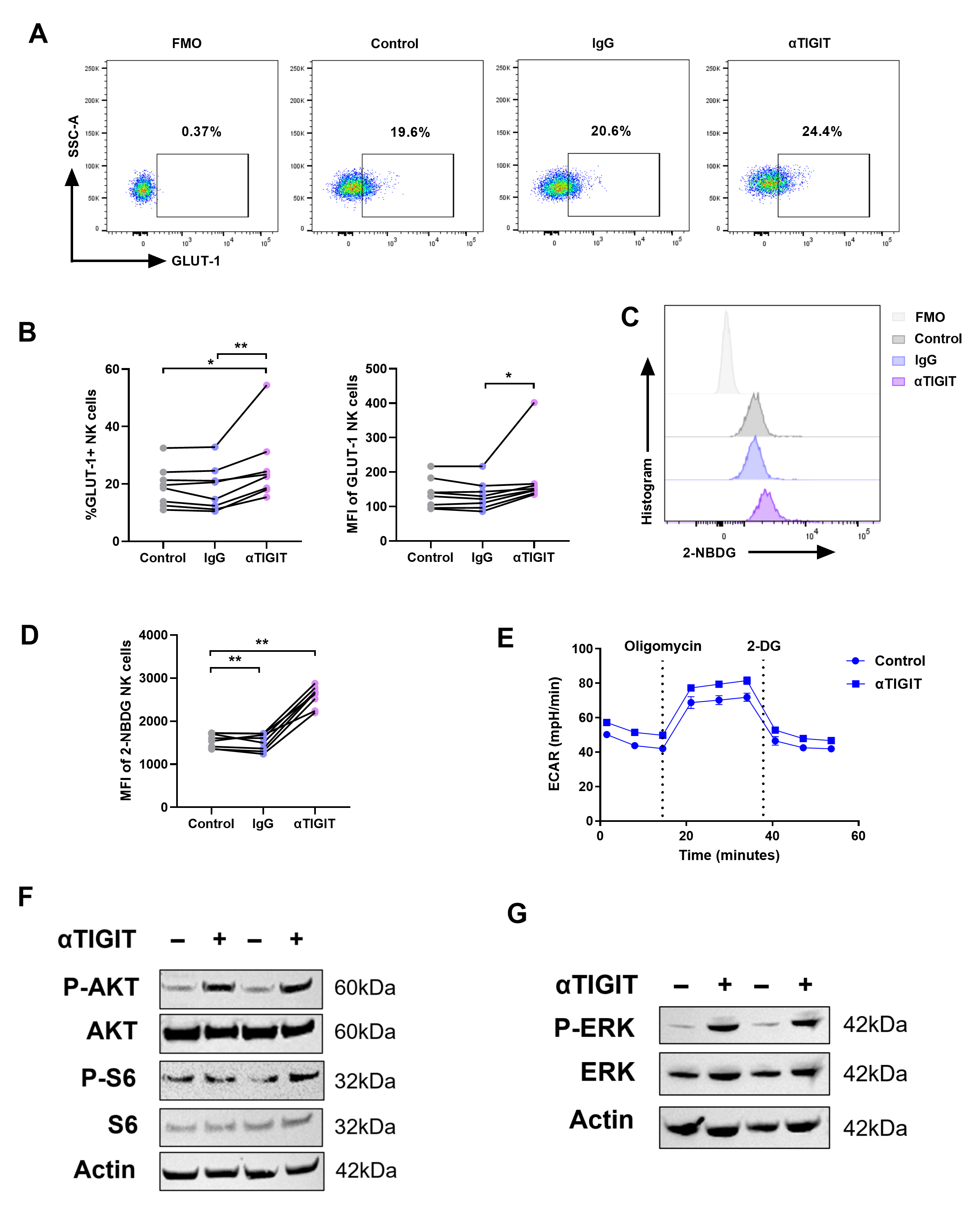

Supplement: Supplementary file 3 — Supplementary Figure 2 [file 41419_2025_8039_MOESM3_ESM.tif]

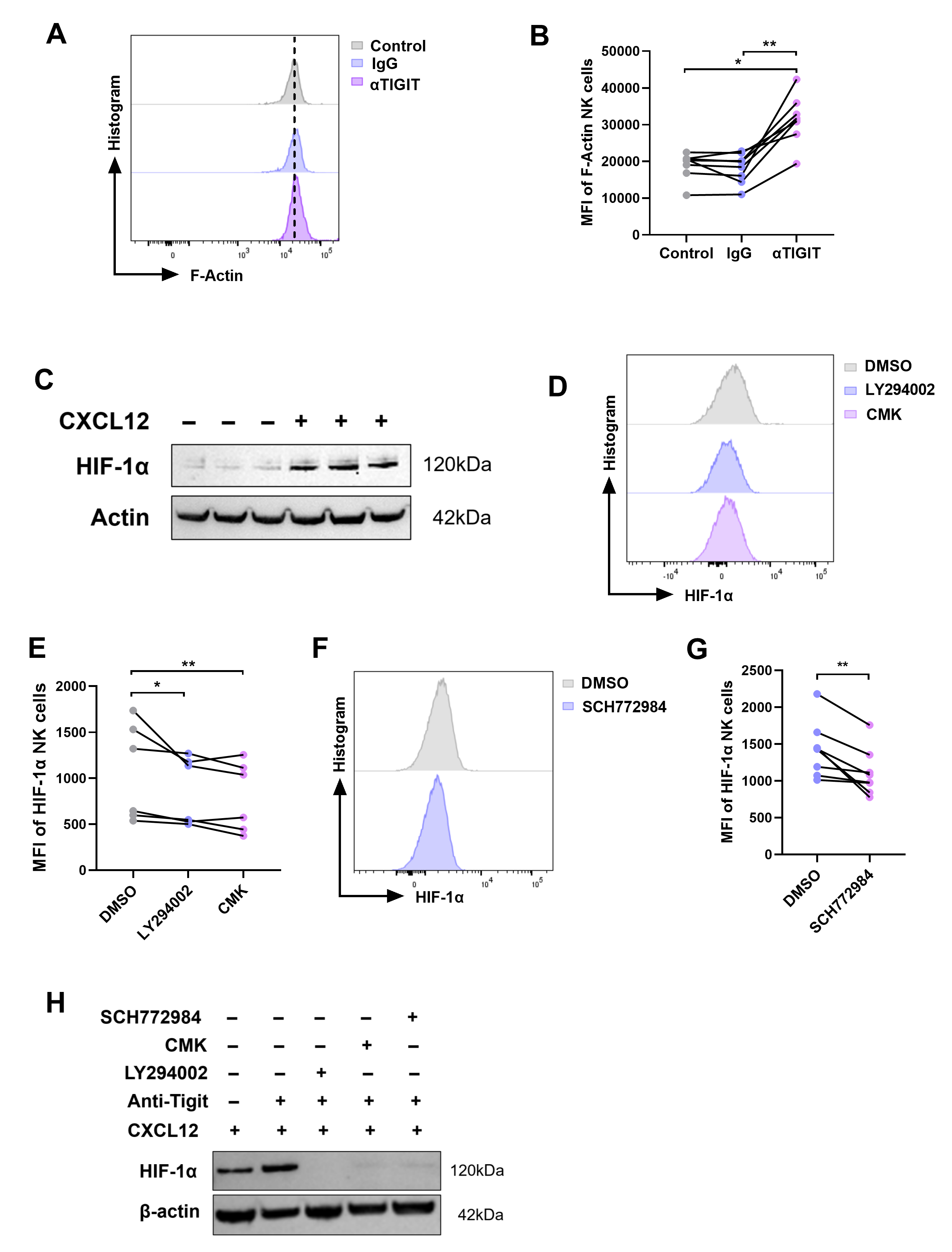

Supplement: Supplementary file 4 — Supplementary Figure 3 [file 41419_2025_8039_MOESM4_ESM.tif]

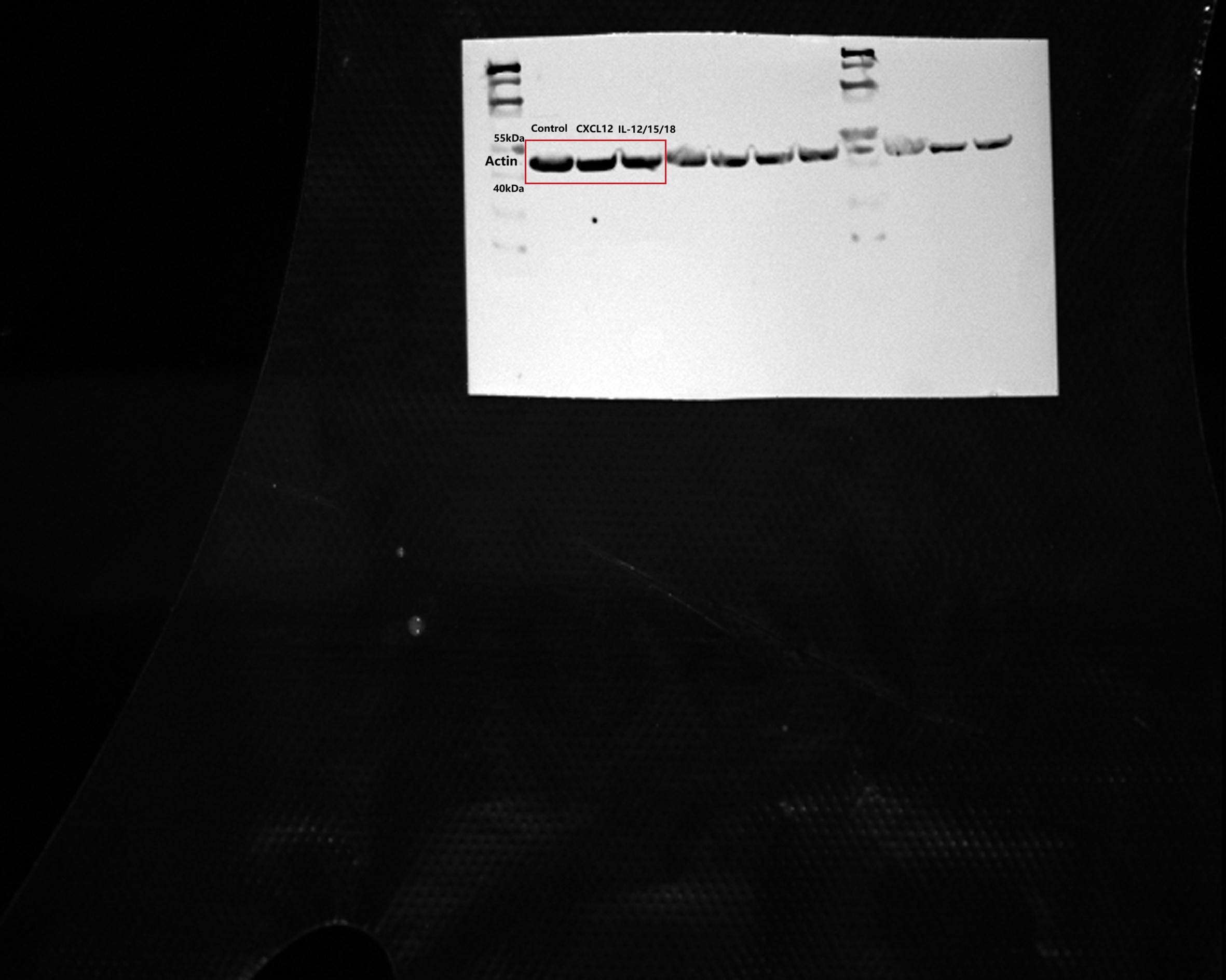

Supplement: Supplementary file 5 — Full and uncropped western blots [file 41419_2025_8039_MOESM5_ESM.zip › Supplementary Materials-Full and uncropped western blots/Supplementary Figure 1 Full and uncropped western blots/Actin.jpg]

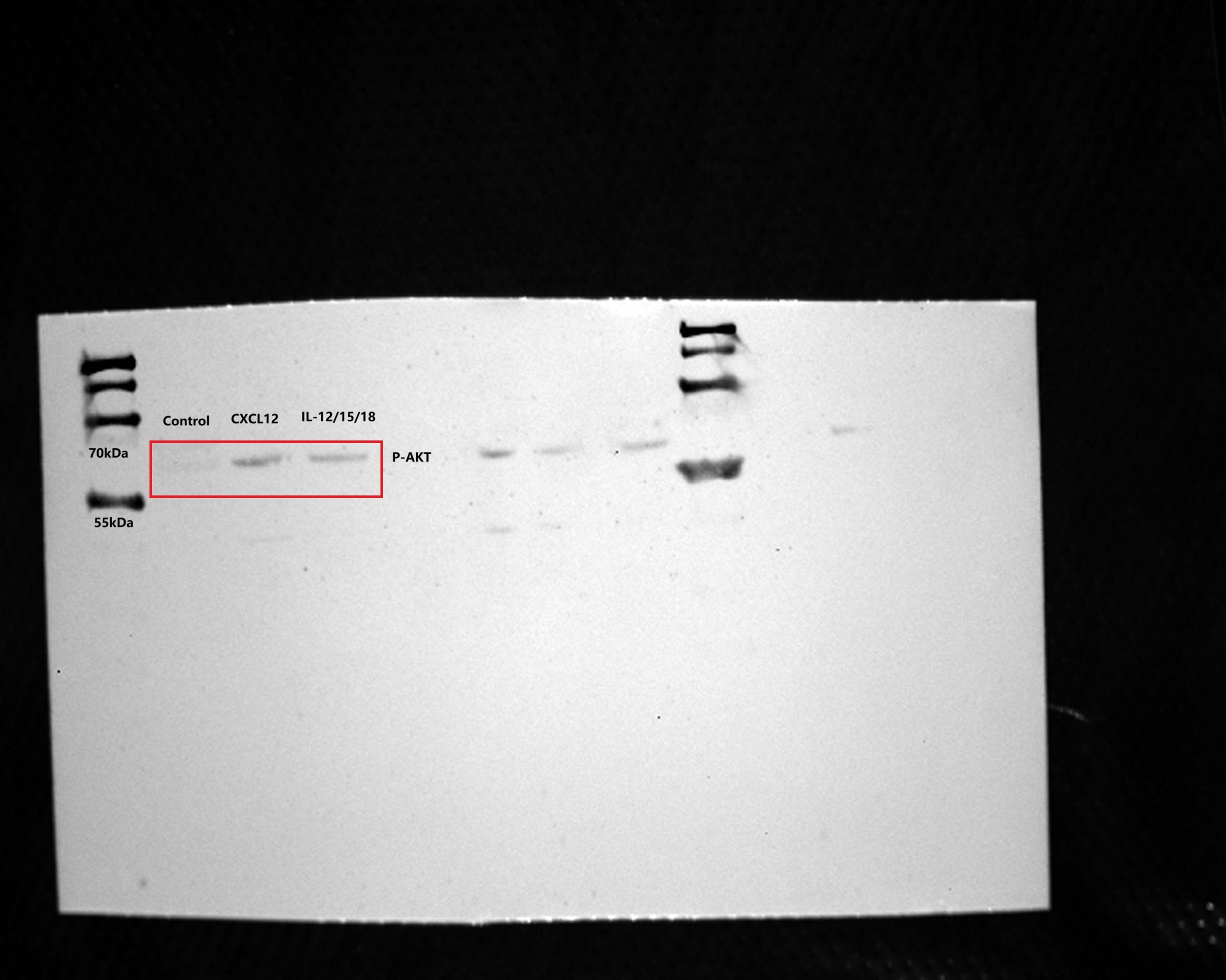

Supplement: Supplementary file 5 — Full and uncropped western blots [file 41419_2025_8039_MOESM5_ESM.zip › Supplementary Materials-Full and uncropped western blots/Supplementary Figure 1 Full and uncropped western blots/p-AKT.jpg]

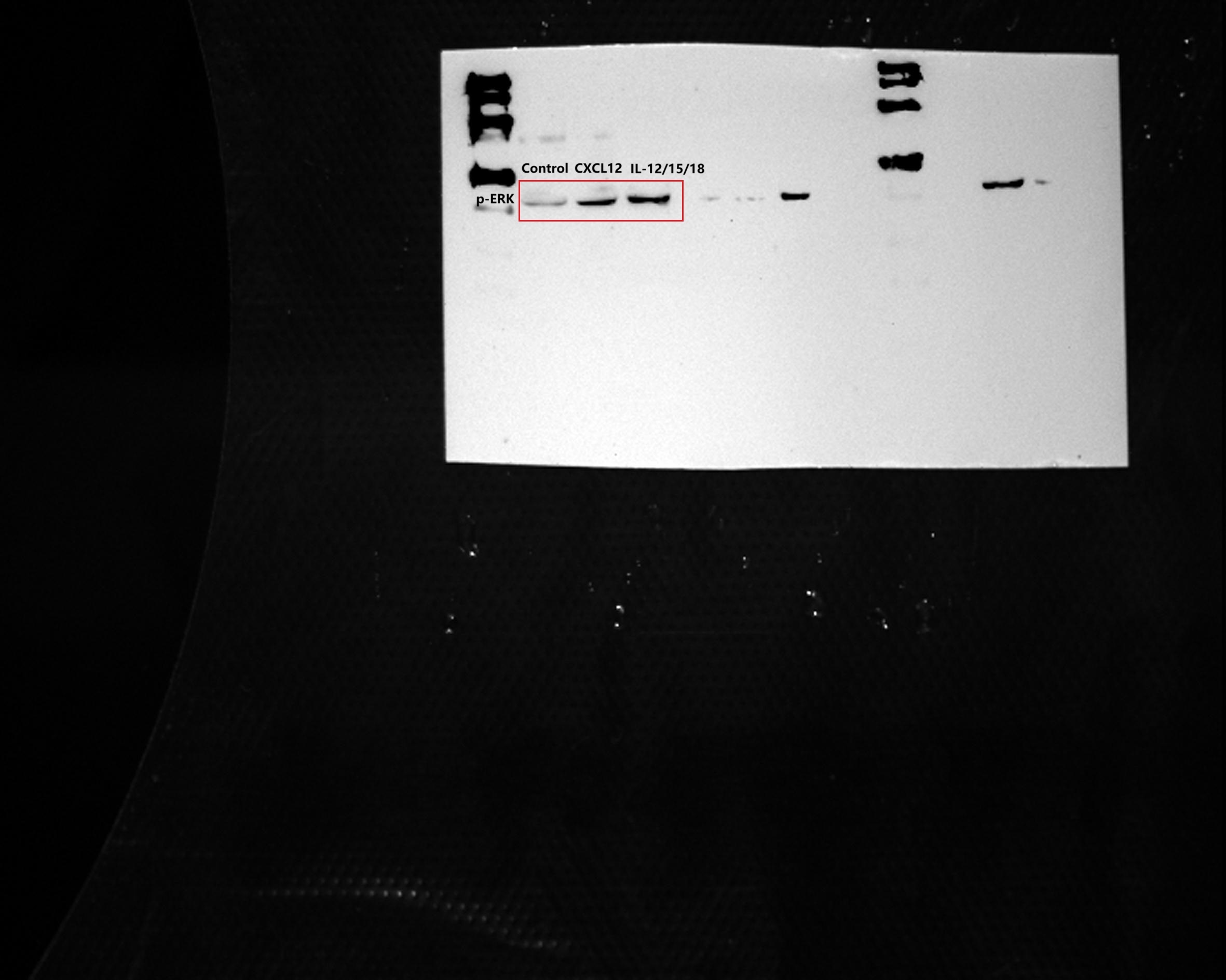

Supplement: Supplementary file 5 — Full and uncropped western blots [file 41419_2025_8039_MOESM5_ESM.zip › Supplementary Materials-Full and uncropped western blots/Supplementary Figure 1 Full and uncropped western blots/p-ERK.jpg]

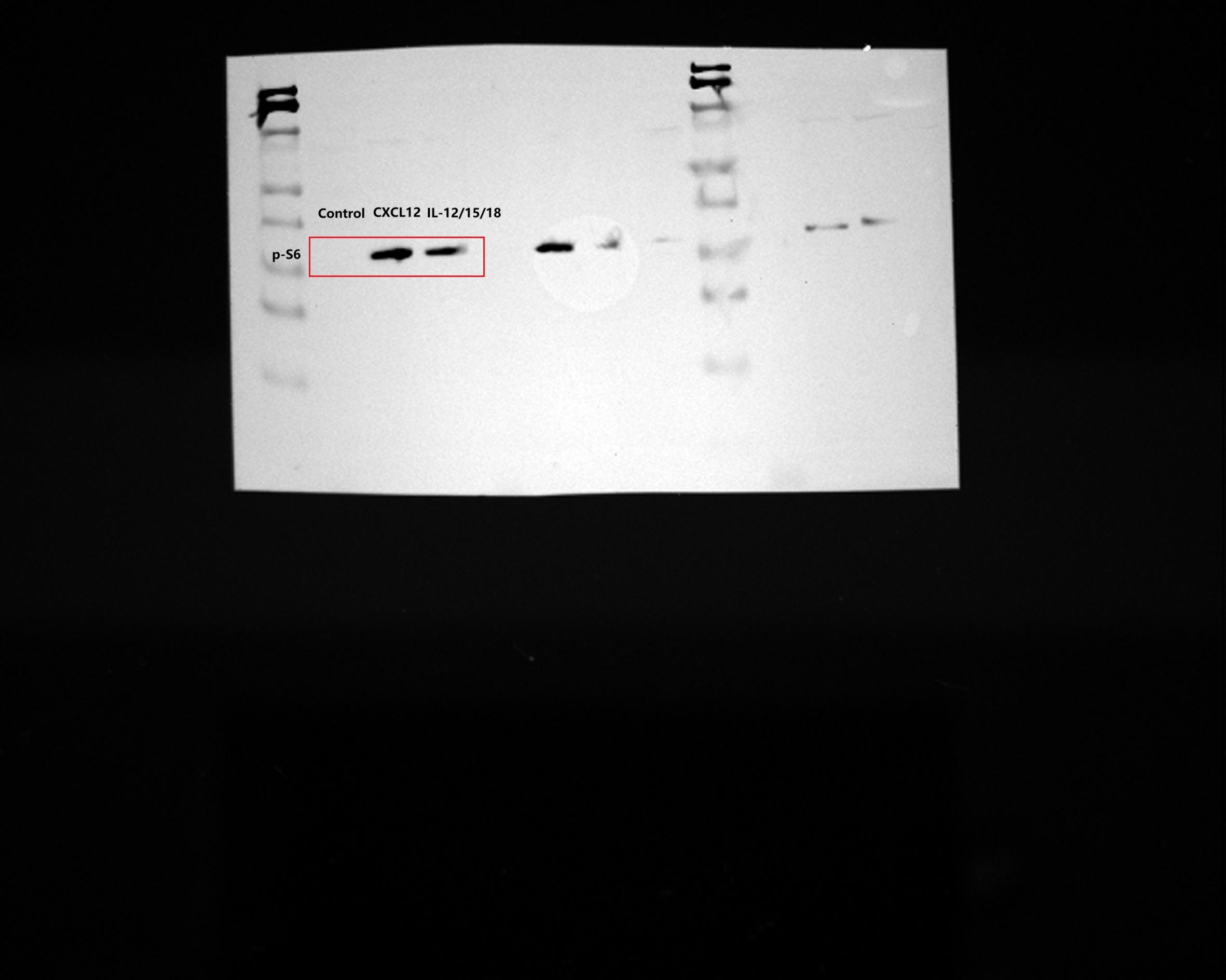

Supplement: Supplementary file 5 — Full and uncropped western blots [file 41419_2025_8039_MOESM5_ESM.zip › Supplementary Materials-Full and uncropped western blots/Supplementary Figure 1 Full and uncropped western blots/p-S6.jpg]

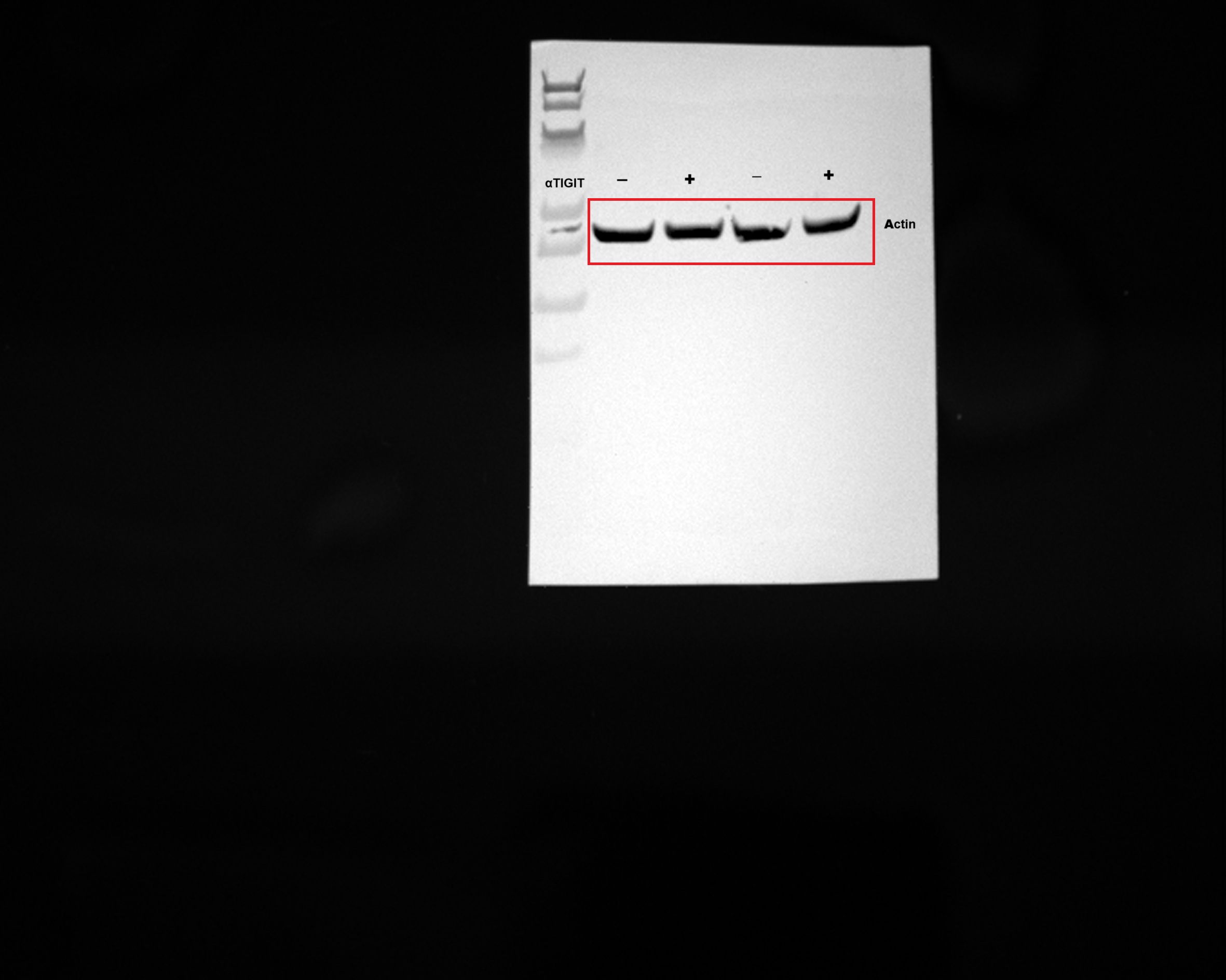

Supplement: Supplementary file 5 — Full and uncropped western blots [file 41419_2025_8039_MOESM5_ESM.zip › Supplementary Materials-Full and uncropped western blots/Supplementary Figure 2 Full and uncropped western blots/Supplementary Figure 2F/Actin 1.jpg]

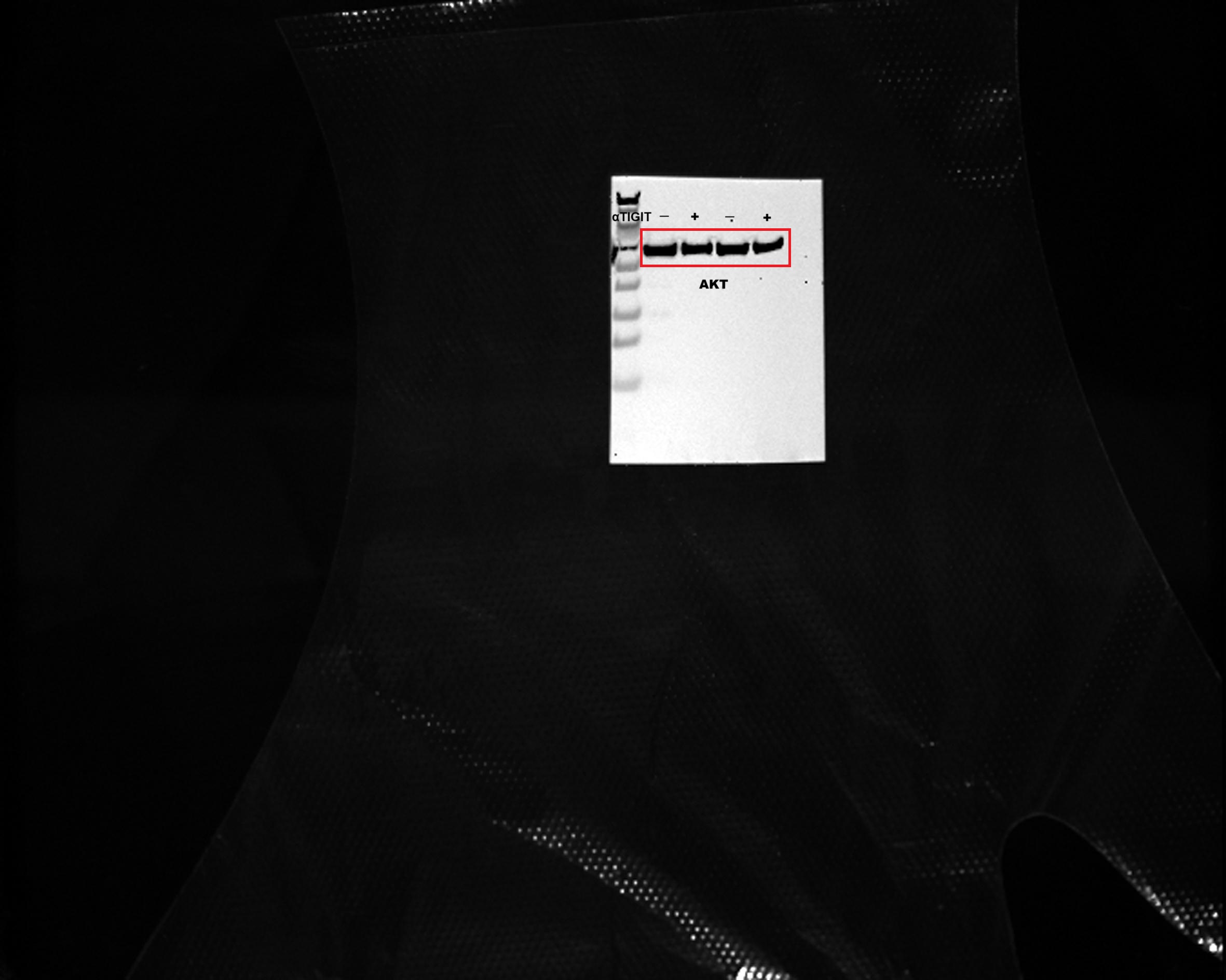

Supplement: Supplementary file 5 — Full and uncropped western blots [file 41419_2025_8039_MOESM5_ESM.zip › Supplementary Materials-Full and uncropped western blots/Supplementary Figure 2 Full and uncropped western blots/Supplementary Figure 2F/AKT.jpg]

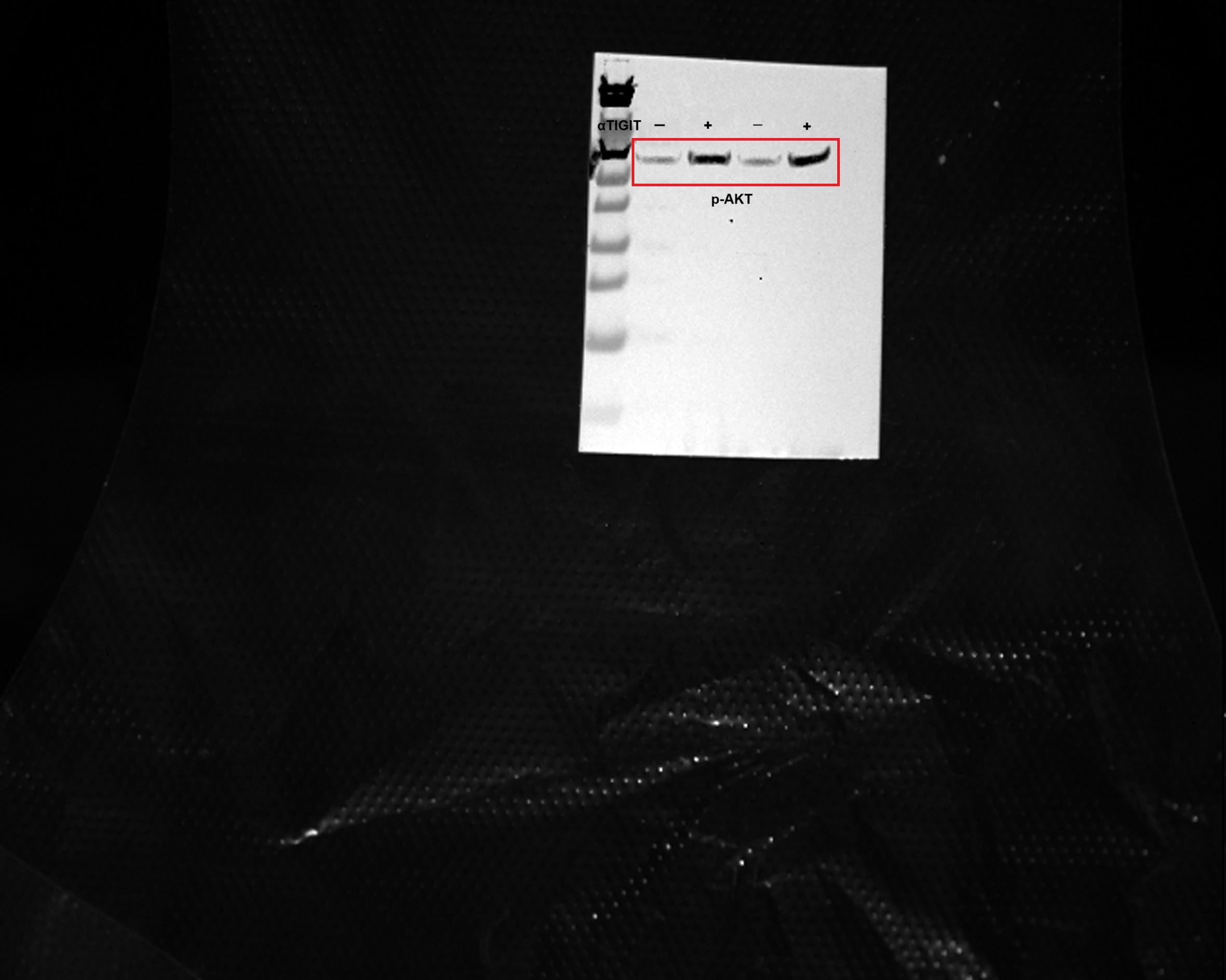

Supplement: Supplementary file 5 — Full and uncropped western blots [file 41419_2025_8039_MOESM5_ESM.zip › Supplementary Materials-Full and uncropped western blots/Supplementary Figure 2 Full and uncropped western blots/Supplementary Figure 2F/p-AKT.jpg]

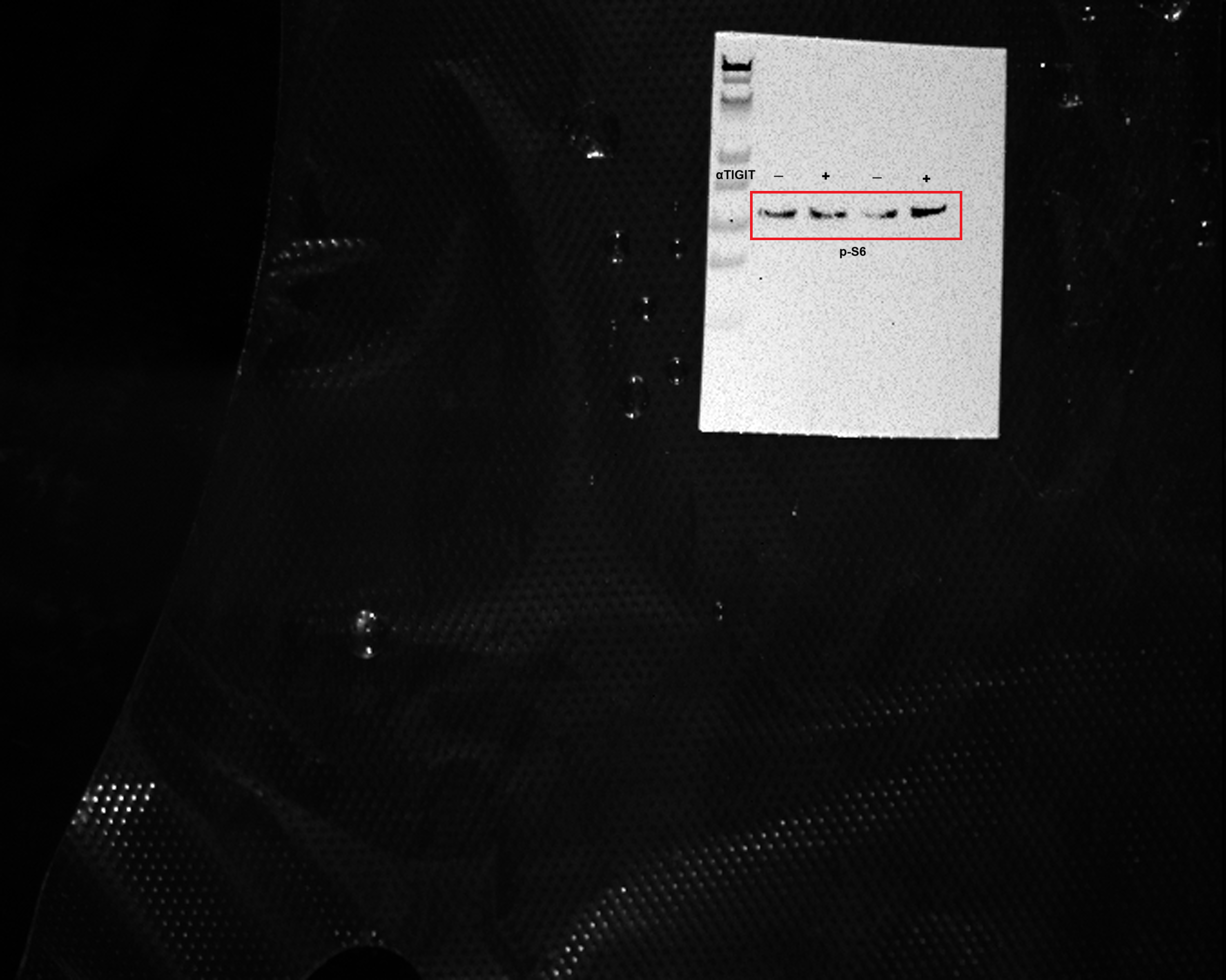

Supplement: Supplementary file 5 — Full and uncropped western blots [file 41419_2025_8039_MOESM5_ESM.zip › Supplementary Materials-Full and uncropped western blots/Supplementary Figure 2 Full and uncropped western blots/Supplementary Figure 2F/p-S6.png]

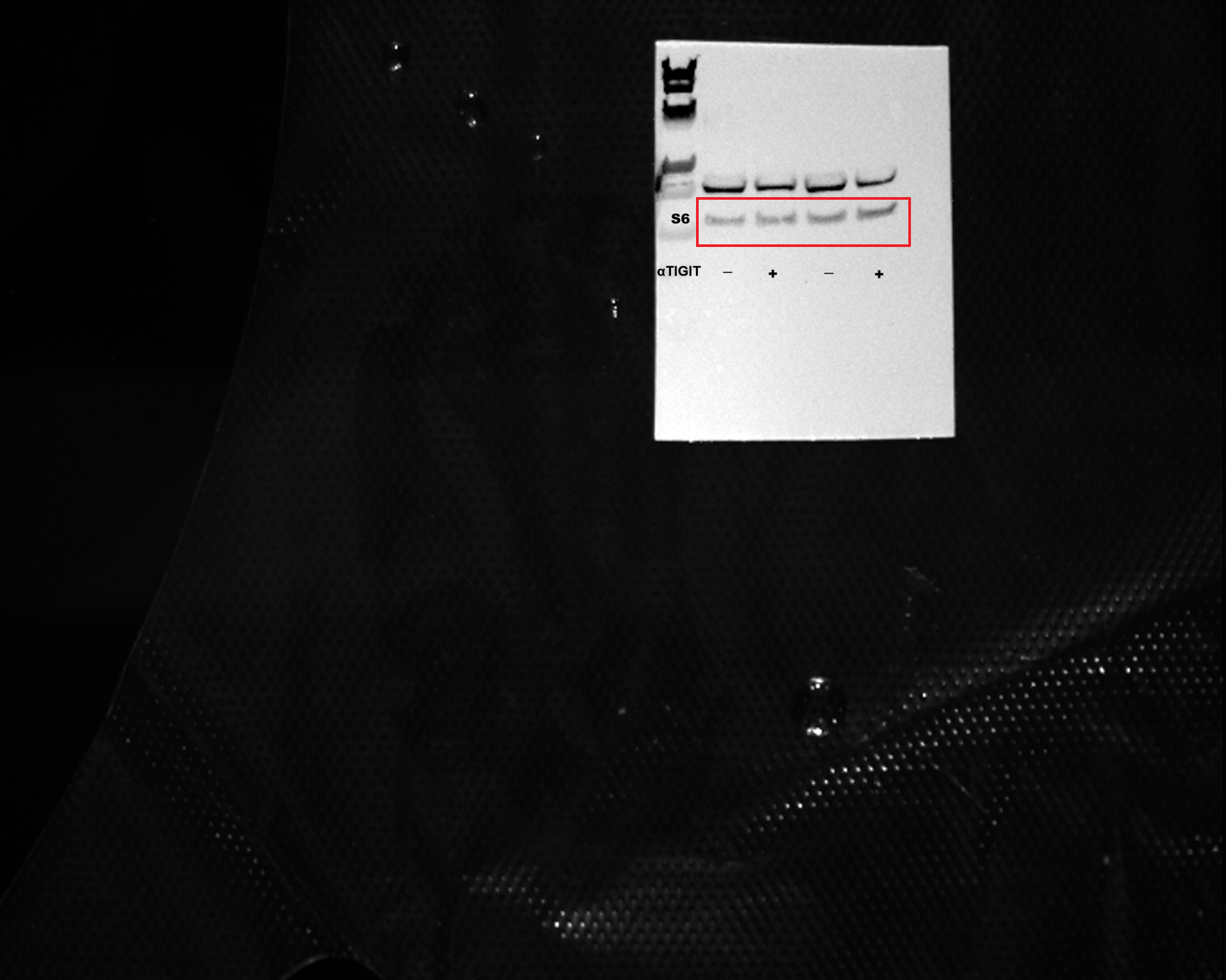

Supplement: Supplementary file 5 — Full and uncropped western blots [file 41419_2025_8039_MOESM5_ESM.zip › Supplementary Materials-Full and uncropped western blots/Supplementary Figure 2 Full and uncropped western blots/Supplementary Figure 2F/S6.png]

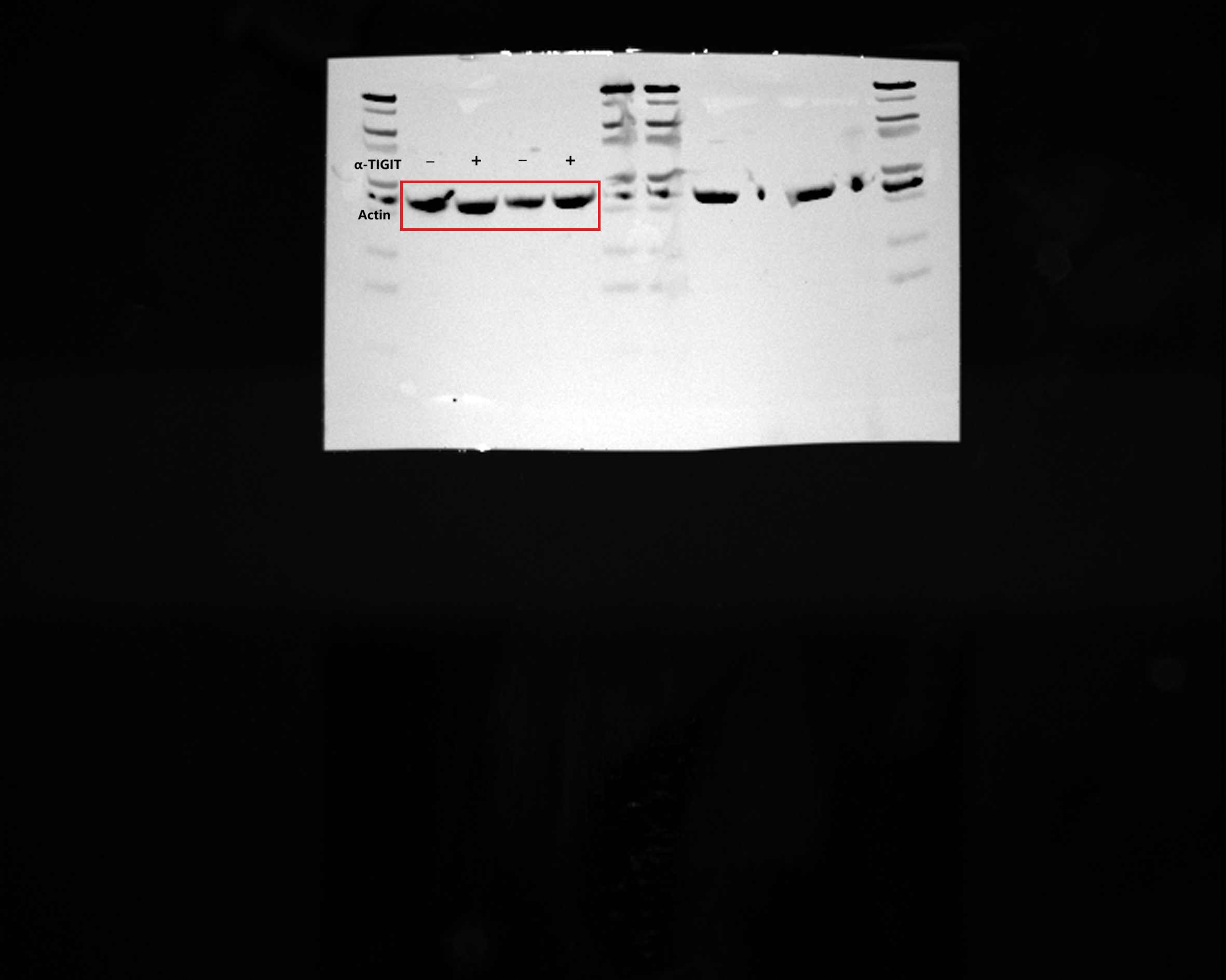

Supplement: Supplementary file 5 — Full and uncropped western blots [file 41419_2025_8039_MOESM5_ESM.zip › Supplementary Materials-Full and uncropped western blots/Supplementary Figure 2 Full and uncropped western blots/Supplementary Figure 2G/Actin.jpg]

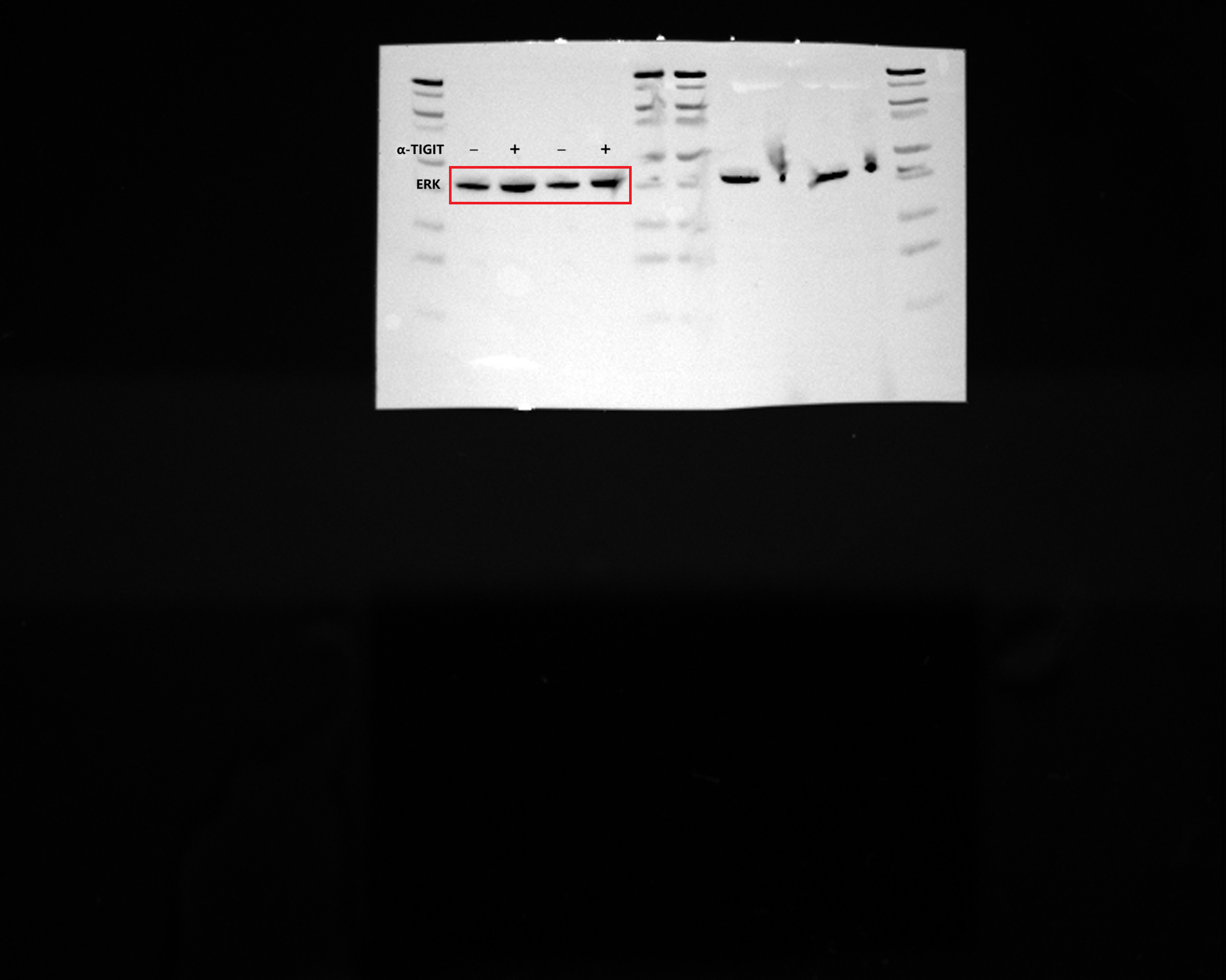

Supplement: Supplementary file 5 — Full and uncropped western blots [file 41419_2025_8039_MOESM5_ESM.zip › Supplementary Materials-Full and uncropped western blots/Supplementary Figure 2 Full and uncropped western blots/Supplementary Figure 2G/ERK.png]

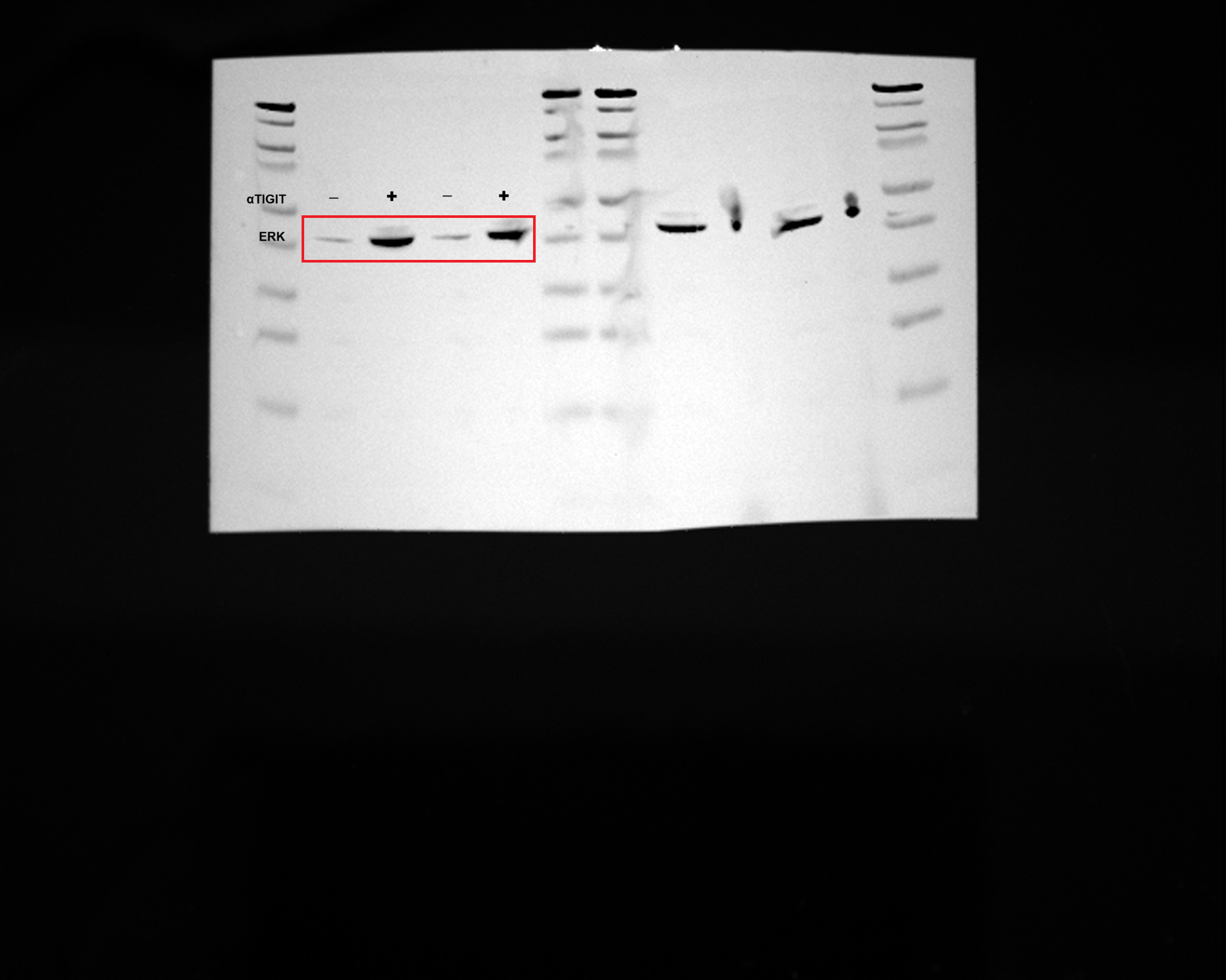

Supplement: Supplementary file 5 — Full and uncropped western blots [file 41419_2025_8039_MOESM5_ESM.zip › Supplementary Materials-Full and uncropped western blots/Supplementary Figure 2 Full and uncropped western blots/Supplementary Figure 2G/p-ERK.png]

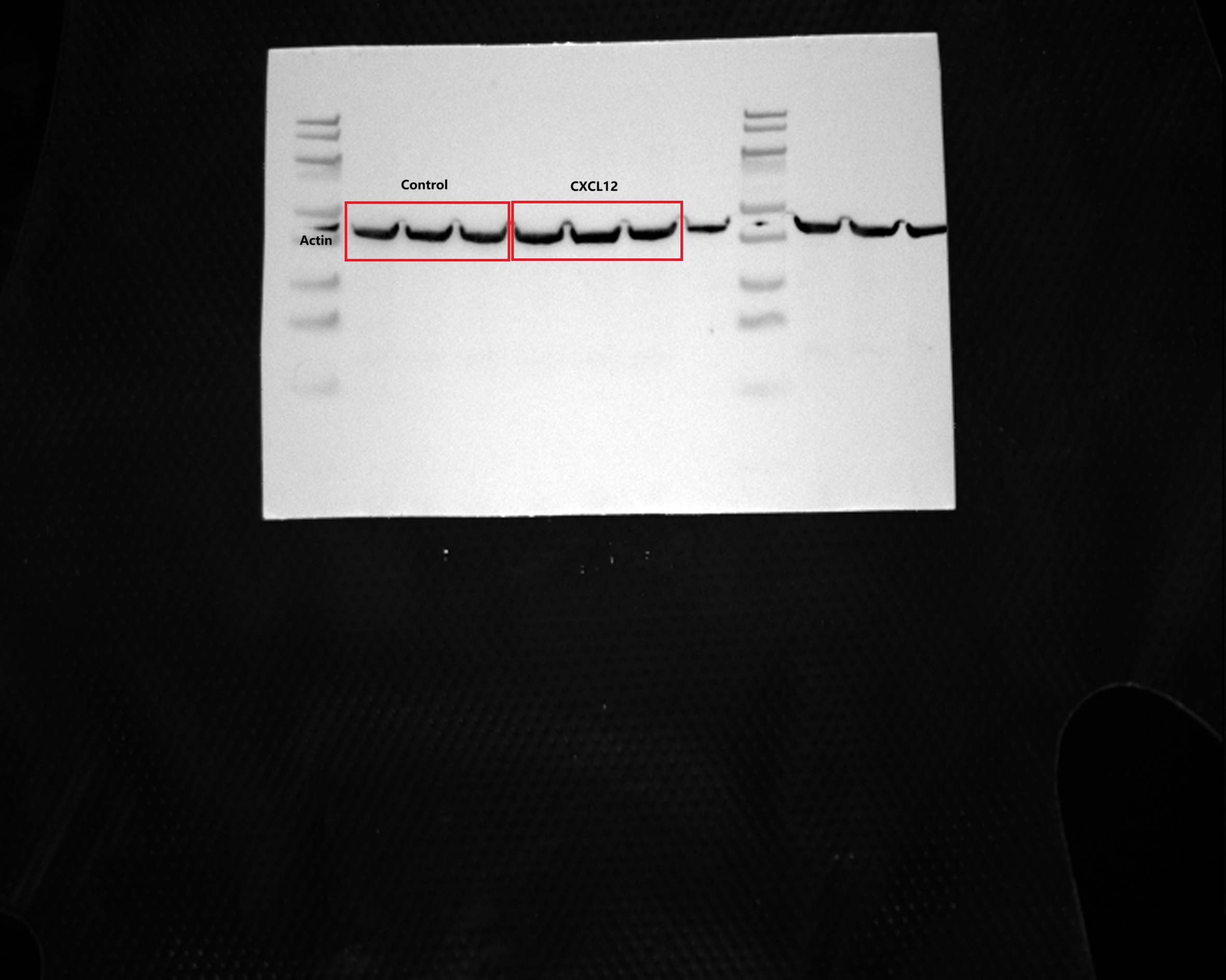

Supplement: Supplementary file 5 — Full and uncropped western blots [file 41419_2025_8039_MOESM5_ESM.zip › Supplementary Materials-Full and uncropped western blots/Supplementary Figure 3 Full and uncropped western blots/Supplementary Figure 3C/Actin.jpg]

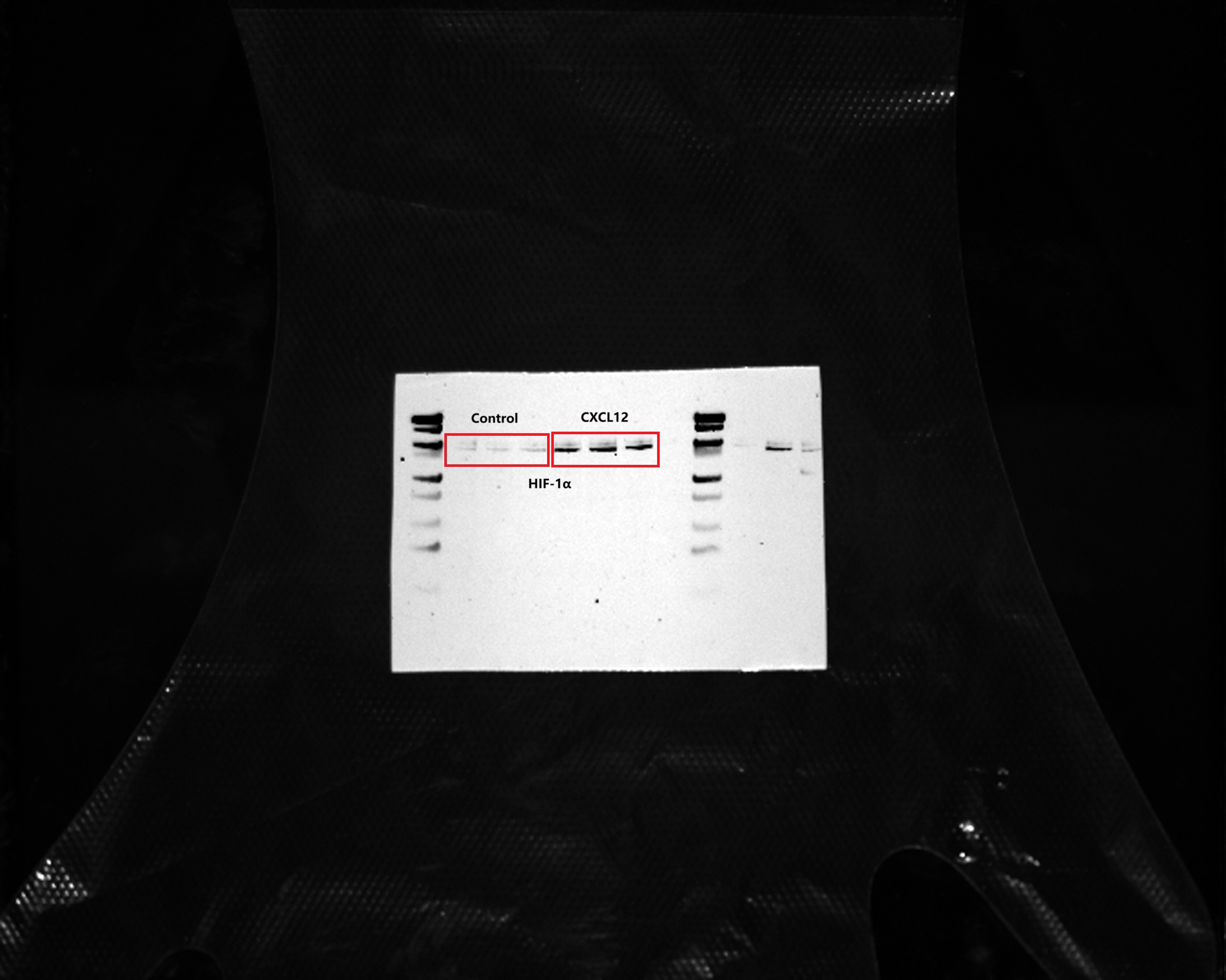

Supplement: Supplementary file 5 — Full and uncropped western blots [file 41419_2025_8039_MOESM5_ESM.zip › Supplementary Materials-Full and uncropped western blots/Supplementary Figure 3 Full and uncropped western blots/Supplementary Figure 3C/HIF-1α.jpg]

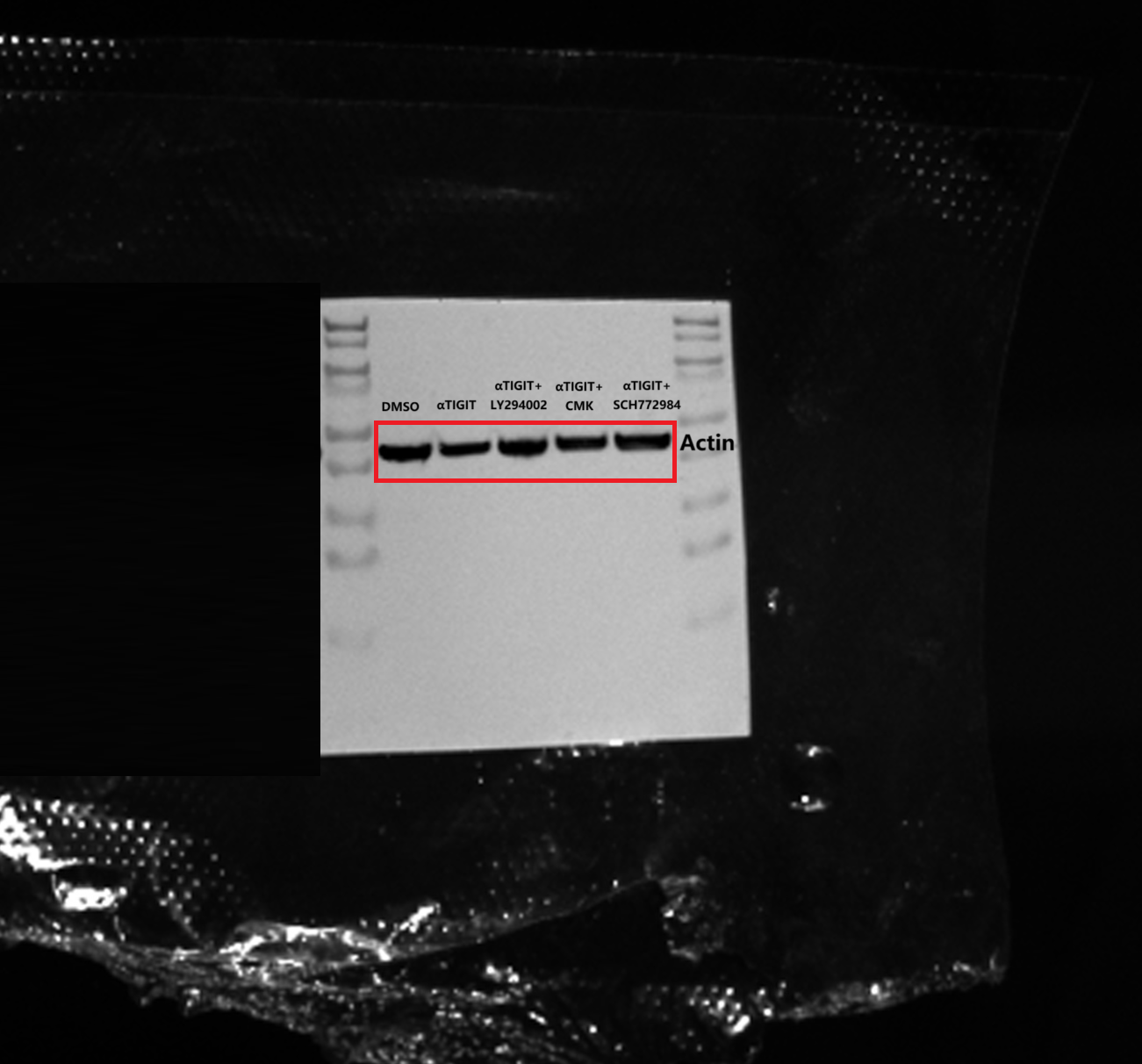

Supplement: Supplementary file 5 — Full and uncropped western blots [file 41419_2025_8039_MOESM5_ESM.zip › Supplementary Materials-Full and uncropped western blots/Supplementary Figure 3 Full and uncropped western blots/Supplementary Figure 3H/Actin.tif]

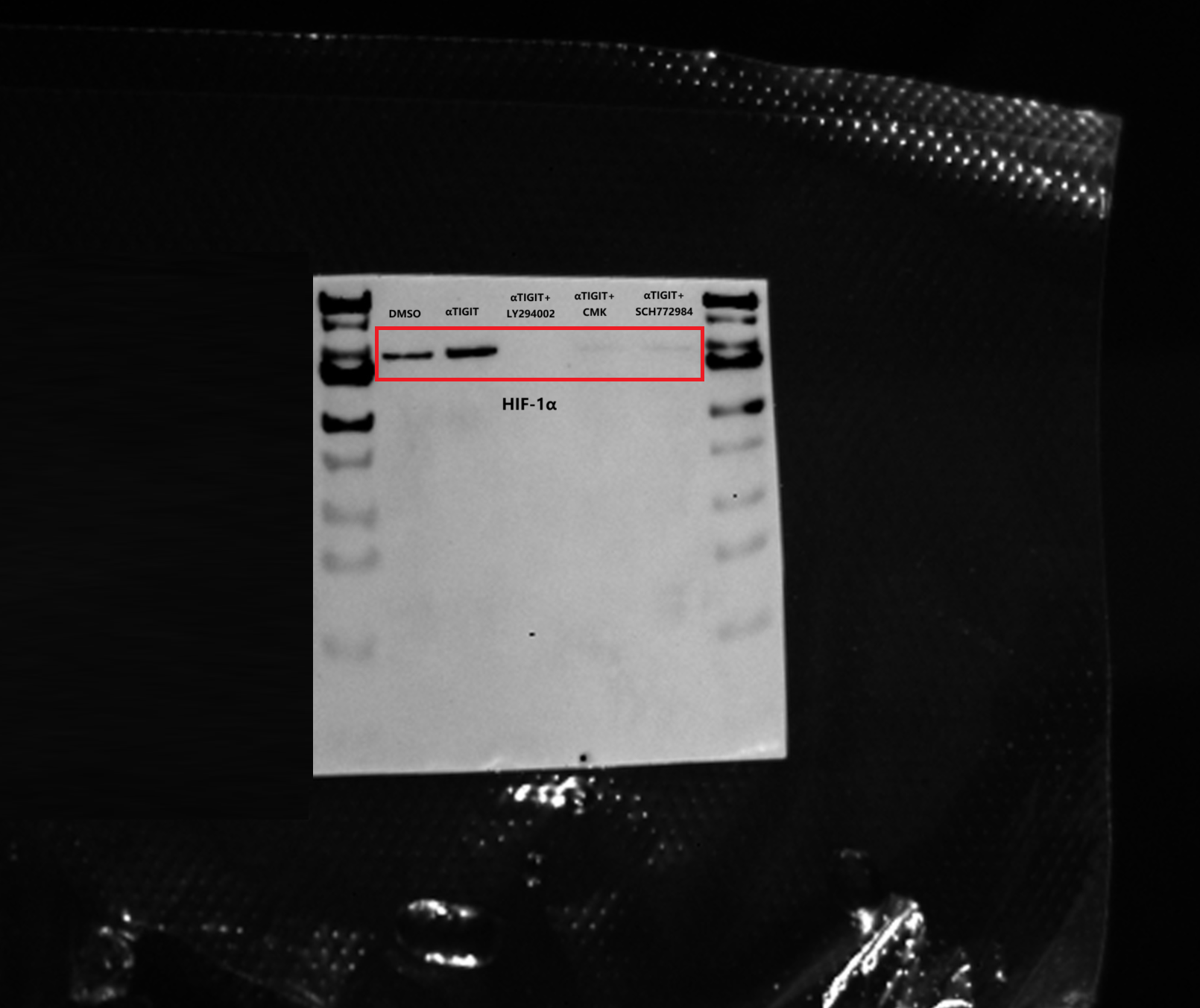

Supplement: Supplementary file 5 — Full and uncropped western blots [file 41419_2025_8039_MOESM5_ESM.zip › Supplementary Materials-Full and uncropped western blots/Supplementary Figure 3 Full and uncropped western blots/Supplementary Figure 3H/HIF-1α.tif]
